# Targeting myeloid suppressive cells revives cytotoxic anti-tumor responses in pancreatic cancer

**DOI:** 10.1016/j.isci.2022.105317

**Published:** 2022-10-09

**Authors:** Dhifaf Sarhan, Silke Eisinger, Fei He, Maria Bergsland, Catarina Pelicano, Caroline Driescher, Kajsa Westberg, Itziar Ibarlucea Benitez, Rawan Humoud, Giorgia Palano, Shuijie Li, Valentina Carannante, Jonas Muhr, Björn Önfelt, Susanne Schlisio, Jeffrey V. Ravetch, Rainer Heuchel, Matthias J. Löhr, Mikael C.I. Karlsson

**Affiliations:** 1 Department of Laboratory Medicine, Division of Pathology, Karolinska Institutet, Stockholm, Sweden, SE-141 521; 2 Department of Microbiology, Tumor and Cell Biology, Karolinska Institutet, SE-171 77 Stockholm, Sweden; 3 Department of Cell and Molecular Biology, Karolinska Institutet, SE-171 77 Stockholm, Sweden; 4 University of Cambridge, Cancer Research UK Cambridge Institute, Cambridge, UK; 5 Department of Pathology, Heinrich-Heine University of Düsseldorf, 40225 Düsseldorf, Germany; 6 Laboratory of Molecular Genetics and Immunology, The Rockefeller University, New York, NY 10065, USA; 7 Department of Medicine, Karolinska University Hospital, Huddinge, Sweden; 8 Department of Applied Physics, Science for Life Laboratory, KTH Royal Institute of Technology, Stockholm, Sweden; 9 Pancreatic Cancer Research Lab, Department of Clinical Science, Intervention and Technology, Karolinska Institutet, SE-141 86 Stockholm, Sweden

**Keywords:** Immunology, Components of the immune system, Cancer

## Abstract

Immunotherapy for cancer that aims to promote T cell anti-tumor activity has changed current clinical practice, where some previously lethal cancers have now become treatable. However, clinical trials with low response rates have been disappointing for pancreatic ductal adenocarcinoma (PDAC). One suggested explanation is the accumulation of dominantly immunosuppressive tumor-associated macrophages and myeloid-derived suppressor cells in the tumor microenvironment (TME). Using retrospectively collected tumor specimens and transcriptomic data from PDAC, we demonstrate that expression of the scavenger receptor MARCO correlates with poor prognosis and a lymphocyte-excluding tumor phenotype. PDAC cell lines produce IL-10 and induce high expression of MARCO in myeloid cells, and this was further enhanced during hypoxic conditions. These myeloid cells suppressed effector T and natural killer (NK) cells and blocked NK cell tumor infiltration and tumor killing in a PDAC 3D-spheroid model. Anti-human MARCO (anti-hMARCO) antibody targeting triggered the repolarization of tumor-associated macrophages and activated the inflammasome machinery, resulting in IL-18 production. This in turn enhanced T cell and NK cell functions. The targeting of MARCO thus remodels the TME and represents a rational approach to make immunotherapy more efficient in PDAC patients.

## Highlights

• Pancreatic tumors drive an immunosuppressive phenotype of myeloid cells
• Antibodies activate MARCO-expressing myeloid cells to release cytotoxic cells
• Expression of MARCO is connected to decreasing survival in human pancreatic cancer

## Graphical abstract

**Figure:**
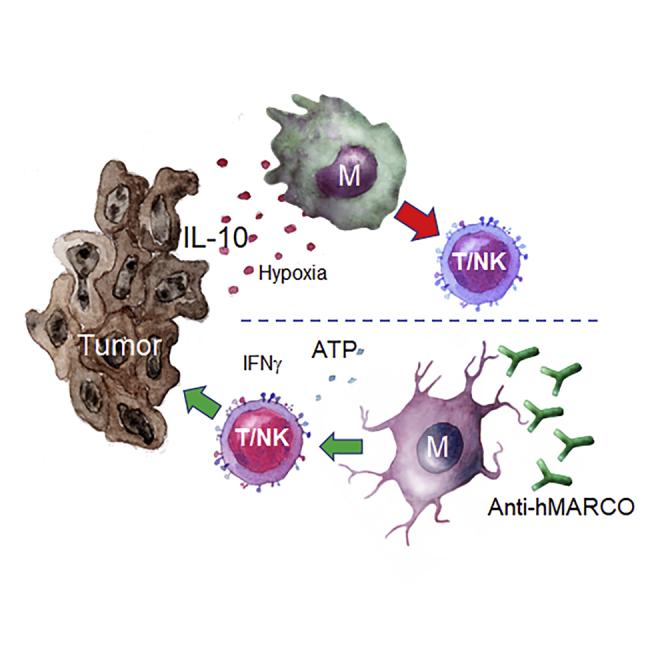

## Introduction

The majority of pancreatic cancer cases are diagnosed as pancreatic ductal adenocarcinoma (PDAC) and are characterized by late diagnosis, inadequate responses to conventional therapies and poor prognosis (Conroy et al., 2011; Siegel et al., 2016; Von Hoff et al., 2013). PDAC tumors have a dense stroma with fibrotic connective tissue that surrounds the tumor, accounting for up to 80% of the tumor volume. This limits the efficiency of drug delivery and creates an immunosuppressed tumor environment (Erkan et al., 2010; Neesse et al., 2011).

Checkpoint immunotherapies such as monotherapy and combination therapy utilizing anti-PD1 and anti-CTLA4 antibodies have been remarkably successful in other cancers but have been ineffective in pancreatic cancer (Brahmer et al., 2012; O'Reilly et al., 2019a; O'Reilly et al., 2019b; Royal et al., 2010). Several possible reasons for these failures have been suggested, including an anti-inflammatory cytokine bias (Schmiechen and Stromnes, 2020) creating an immunosuppressive tumor microenvironment (TME) characterized by poor infiltration and activation of T and natural killer (NK) cells and a prominent myeloid cell presence (Clark et al., 2007; Stromnes et al., 2017). Tumor-associated macrophages (TAMs) and myeloid-derived suppressor cells (MDSCs) contribute to the major immunosuppressive component of the stroma in many tumors, and increased numbers of these cells are associated with poor prognosis in pancreatic cancer (Mantovani et al., 2006, 2009). They produce several factors regulating inflammation and tissue homeostasis that allow for tumor growth and metastasis, such as TGFβ, VEGF, IL-10, and reactive oxygen species (ROS). During tumor progression, processes such as myeloid cell transition from pro-inflammatory to anti-inflammatory states are dominating, resulting in the generation of an immunosuppressive TME (Kiss et al., 2018). This macrophage polarization depends on several factors in the TME including cytokine milieu and potential triggers of sterile inflammatory responses. Examples of these are factors released by dying cells or stressed cells including extracellular ATP (Gombault et al., 2012). For ATP the response is context dependent and can go in both pro- and anti-inflammatory directions (Faas et al., 2017) depending on the combination of signals.

In this context there is much current research effort to identify combinatory treatment alternatives that target the myeloid compartment in the TME (Johnson et al., 2017). We previously demonstrated in mice that targeting the scavenger receptor (SR) MARCO (macrophage receptor with collagenous structure) in experimental melanoma, colon and breast cancer mouse models reduces tumor growth and impairs metastasis in breast cancer (Georgoudaki et al., 2016). We also demonstrated that MARCO-expressing macrophages are present in human breast cancer, malignant melanoma, periampullary adenocarcinoma, and lung cancer (Georgoudaki et al., 2016; La Fleur et al., 2018). Since MARCO is conserved between humans and mice, we hypothesize that targeting MARCO in humans could remodel the suppressive environment and reduce the anti-tumor responses to increase the efficacy of immunotherapy in PDAC. In this study, we investigated the presence of MARCO-expressing myeloid cells in PDAC and their role in orchestrating immune cell responses in the TME. We determined that MARCO is an independent prognostic factor and that increased MARCO expression in PDAC tissues correlates with poor tumor infiltration of cytotoxic T cells and NK cells. Furthermore, we developed antibodies against human MARCO and observed that anti-human MARCO (anti-hMARCO) treatment induced extracellular ATP release, inducing NLRP3 activation and subsequently IL-18 expression which is important for effector cell function. The targeting of MARCO is thus similar between mice and humans and modulates the immunosuppressive effects of myeloid cells. Our findings demonstrate a possible approach to specifically target suppressive myeloid cells for the treatment of PDAC.

## Results

### PDAC are heavily infiltrated by MARCO-expressing TAMs, and the presence of these predicts poor survival

**Figure 1:**
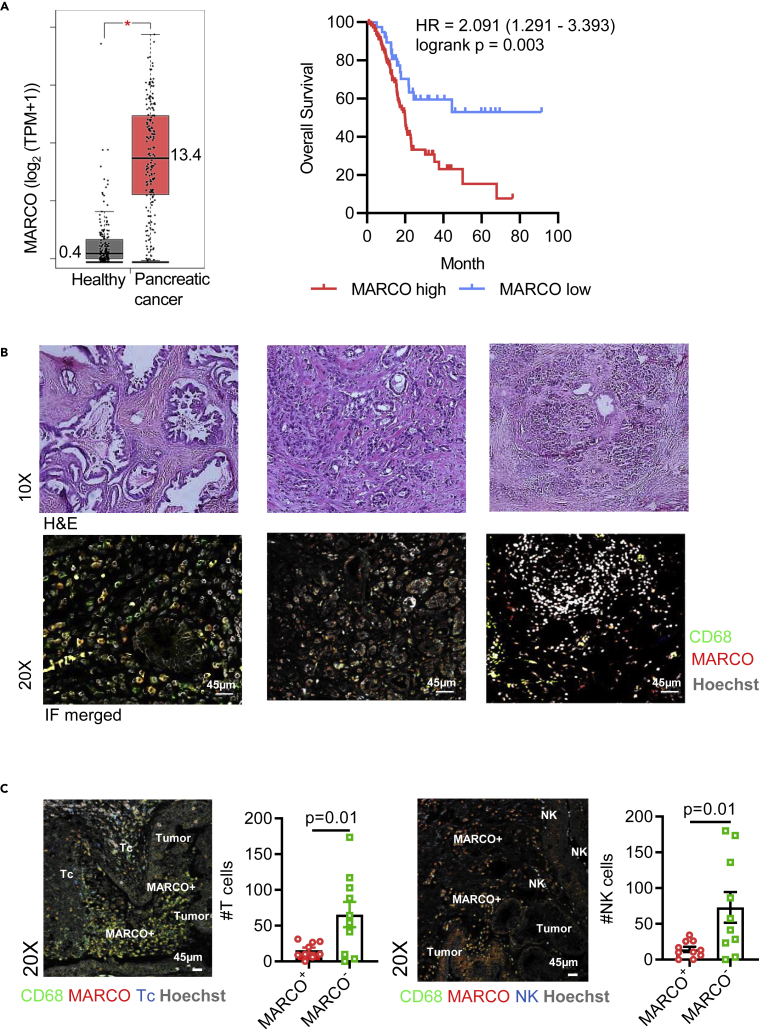
MARCO expression in human pancreatic cancer (A) Left panel: Analysis of mRNA expression of MARCO in 171 healthy individuals and 150 PDAC samples. Log_2_, TPM+1 are shown. Right panel: Kaplan-Meier plot of PDAC patients based on low versus high MARCO expression. Log rank statistical analysis was used. (B) Representative hematoxylin and eosin and immunofluorescence staining of snap frozen and paraffin embedded PDAC sections (n = 10). Three different tumors are depicted. (C) Immunofluorescence staining of paraffin-embedded PDAC sections (n = 10) highlighting tumor, MARCO, T cell (**left**), and NK cell areas (**right**). MARCO (red), CD68 (green), CD3 or CD56 (blue), and Hoechst (gray) are shown. Quantification of number of (#) T cells and NK cells in MARCO^+^ and MARCO^−^ macrophage areas in imaged PDAC sections was performed in ImageJ. Every dot represents an average of all imaged areas of one patient. Statistical analyses were performed using student unpaired T-test.

We have previously reported that targeting MARCO-expressing macrophages in murine cancer models reduced tumor growth and impaired metastasis (Georgoudaki et al., 2016). In the current study, we investigated whether MARCO is a marker for human PDAC tumor progression and its potential as a therapeutic target. We first evaluated the prognostic value of MARCO expression in PDAC using the TheCancerGenomeAtlas (TCGA) database. Analysis of bulk RNA sequences revealed a high expression of MARCO in whole tissue of human PDAC, and at higher levels in tumors than in normal pancreas tissue (n = 150 vs. n = 171, 13.4 vs. 0.4 relative transcripts per million [TPM], p < 0.01) (Figure 1A, left panel). Furthermore, a Kaplan-Meier survival analysis of PDAC patients revealed a significant association of high MARCO expression with a worse survival rate (n = 150, p = 0.003, Figure 1A, right panel) and validated in another cohort (Figure S1A), suggesting MARCO as an independent prognostic factor for these patients. Immunohistochemical analyses of tissues from a cohort of PDAC patients confirmed a high prevalence of MARCO-positive cells in the tumor stroma that also co-expressed the pan-macrophage marker CD68 in the majority of investigated specimens (n = 10) (Figures 1B, S1B, and S1C). This confirms that MARCO is expressed in human PDAC and that presence of TAMs expressing this scavenger receptor correlates with poor clinical prognosis.

High infiltration of CD8^+^ T cells is correlated with better prognosis in PDAC, while macrophage and MDSC infiltration is negatively correlated with survival Therefore, we studied the co-localization of MARCO^+^ cells with immune effector cells in paraffin-embedded sections of PDAC tissues. We observed that MARCO^+^ myeloid cells were primarily located in the tumor areas, while T and NK cells were present but mostly excluded from the tumor areas. Moreover, T and NK cells were evident in areas with little or no MARCO expression. Quantification of PDAC tumor specimens revealed a decrease in T and NK cell numbers in the presence of MARCO^+^ macrophage infiltration and higher immune effector infiltration in MARCO^−^ regions (Figure 1C). Overall, our results demonstrate that the presence of MARCO^+^ macrophage in tumors coincides with less T cell and NK cell infiltration. This suggests that the presence of MARCO^+^ myeloid cells can be a measure of a lymphocyte-excluded tumor, sometimes described as a ‘cold tumor phenotype’.

### Pancreatic-cancer-conditioned macrophages express MARCO and have an immunosuppressive phenotype

**Figure 2:**
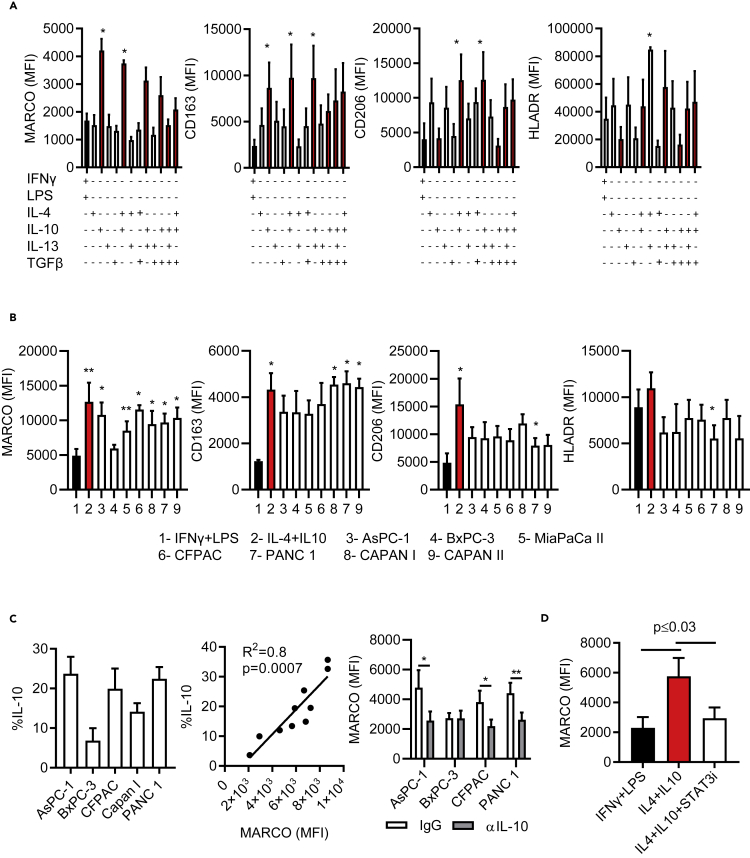
Characterization of human MARCO^+^ myeloid cells (A) Purified monocytes from HD were differentiated in M-CSF and later polarized with combinations of different cytokines including; LPS + IFNγ (black bars), IL-4, IL-10 (red bars), IL-13, and TGFβ. Cells were assessed for typical anti/pro-inflammatory myeloid cell markers and analyzed by flow cytometry. (B) Flow cytometry analysis of macrophages polarized with LPS + IFNγ or IL4+IL10, alternatively co-cultured with different PDAC cell lines: AsPC-1, MiaPaCa II, BxPC-3, CAPAN2, CFPAC, PANC1, and CAPAN I. (A and B) Pooled data (n = 4) are shown as Mean ± SEM mean fluorescence intensity (MFI). Statistical analyses were done using one-way ANOVA comparing all groups to IFNγ+LPS. ∗p ≤ 0.05, ∗∗p ≤ 0.001. (C) PDAC cell lines were cultured in the presence of a protein transport inhibitor for 9 h to assess intracellular IL-10 by flow cytometry. Correlation analysis is shown of the 5 PDAC cell lines IL-10 production and myeloid cell expression of MARCO following co-culture with PDAC cell lines. Linear regression analysis was applied for the statistical analysis of 2 pooled experiments. Myeloid cells were co-cultured with PDAC cell lines in transwell inserts for 48 h in the presence of control IgG or neutralizing antibodies against IL-10 and assessed for MARCO expression. Statistical analyses were done using a student paired T-test. ∗p ≤ 0.05, ∗∗p ≤ 0.001. (D) Flow cytometry analysis of MARCO macrophages polarized with cytokines in the absence or presence of STAT3 small molecule inhibitor. Pooled data (n = 4) are shown as Mean ± SEM, and statistical analyses were performed using paired student T-tests.

Given the accumulation of MARCO-expressing cells in a TME and the association with poor survival in PDAC patients we postulated that MARCO^+^CD68-expressing cells are immunosuppressive macrophages in humans. To investigate this, we next studied the phenotype and function of these cells, through first differentiating human CD14^+^ myeloid cells (monocytes) with M-CSF followed by polarization of the cells with different cytokine cocktails to generate subsets with high or low expression of MARCO. Exposure to IL-10 alone or in combination with IL-4, IL-13, or TGFβ led to increased expression levels of MARCO compared to macrophages polarized with LPS/IFNγ. In addition, the cells co-expressed CD163 and exhibited reduced levels of co-stimulatory molecules and MHCII (HLADR). Stimulation with IL-10 alone induced MARCO expression but was insufficient to promote the expression of mannose receptor-1 (MCR-1/CD206), which is characteristic of immunosuppressive macrophages. Conversely, IL-10 in combination with IL-4 induced CD206 expression (Figures 2A, S1D, and S1E). Therefore, for subsequent experiments we used IL-4 and IL-10 to induce MARCO-expressing immunosuppressive macrophages to study their function.

To investigate whether PDAC tumor cells were able to promote MARCO expression, we co-cultured naive macrophages with PDAC cell lines with different characteristics (Table S1) and assessed their phenotype and function. Cells were separated using transwell inserts to prevent cell-to-cell contact for 48 h, allowing for soluble factors to pass freely. In the cultures where polarization of macrophages was affected by the PDAC lines (TCMs), we recorded similar levels of MARCO and CD163 expression compared to macrophages polarized with IL-10 (Figures 2B and S1F). In addition, these TCMs expressed low HLADR. This led us to the hypothesis that the cell lines might produce IL-10. Flow cytometric analysis confirmed that MARCO expression was only highly induced by the cell lines that produced IL-10. Increased tumor-derived IL-10 also correlated with higher MARCO expression in TCMs, and this was reversed by neutralizing antibodies to IL-10 (Figure 2C). We conclude that pancreatic cells support polarization of macrophages toward a MARCO-expressing subtype. Interestingly, IL-10 expression was found to be correlated with MARCO expression in the PDAC cohort used in this study (Figure S1G). We next addressed the downstream signaling pathways that induce MARCO expression. Given that IL-10 regulates inflammatory responses in macrophages through activation of the signal transducer and activator of transcription-3 (STAT3) (Murray, 2005), we examined the effect of STAT3 on MARCO expression. We specifically inhibited STAT3 by targeting with a small-molecule inhibitor in macrophages during overnight polarization with IL-4 and/or IL-10 and evaluated MARCO expression as a readout. We determined that inhibiting STAT3 resulted in downregulation of MARCO to low levels similar to those on macrophages polarized with LPS/IFNγ (Figure 2D). This was also evident for other markers including ARG I, CD206, CD163 and CD86 as determined by flow cytometry (Figure S1H). The MARCO promoter has several potential sites for binding of STAT3. To investigate binding we used a CHIP-qPCR assay and found repeatedly that STAT3 can directly bind to one of these sites in the MARCO promoter (Figure S1I). These data support a critical role for IL-10 and the STAT3 signaling pathway in PDAC-induced polarization of MARCO-expressing immunosuppressive human macrophages.

### Pancreatic-cancer-conditioned macrophages exhibit MDSC-like features that are amplified by hypoxic conditions

**Figure 3:**
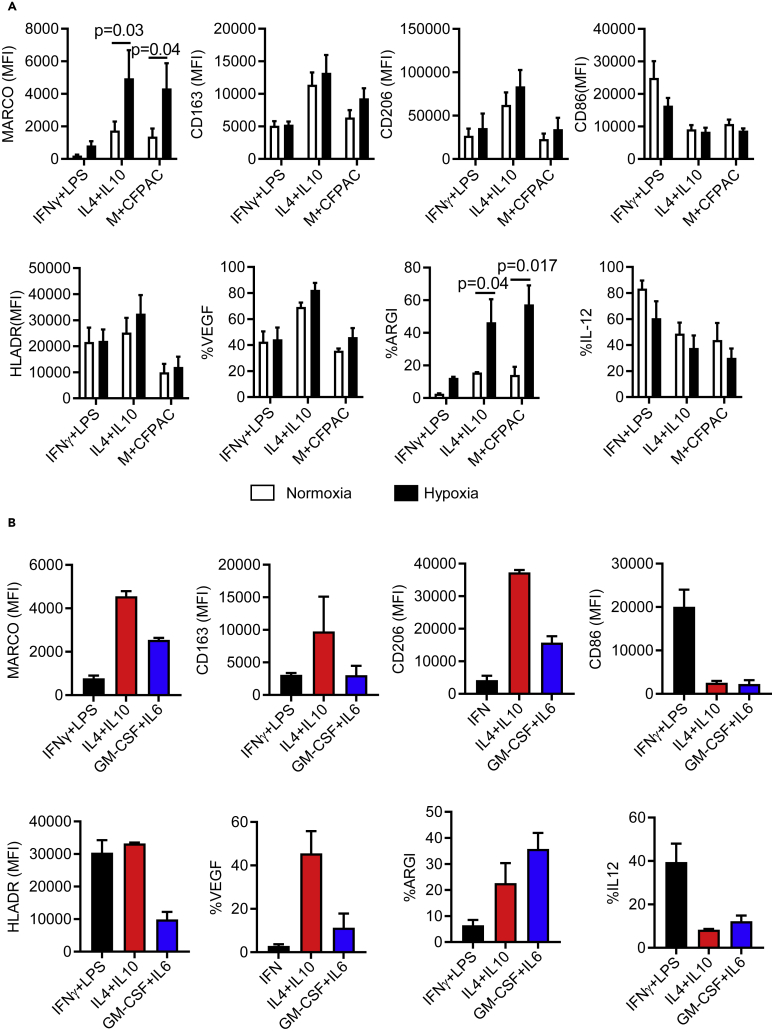
PDAC-conditioned myeloid cells acquire MDSC-like phenotype (A) Myeloid cells polarized with indicated cytokines or conditioned with PDAC cell line CFPAC in transwell co-cultures and incubated overnight in 1% oxygen (hypoxia) or 21% oxygen (normoxia) then analyzed for the expression of different pro vs. anti-inflammatory markers by flow cytometry. (B) Myeloid cells differentiated with indicated cytokines or IL-6+IL-10 to generate MDSC and analyzed for the indicated markers by flow cytometry. Pooled data are shown as mean ± SEM - isotype control.

Hypoxia is one of the hallmarks of the TME in many cancers and causes immune modulation that affects macrophage polarization (Henze and Mazzone, 2016). The central areas of advanced pancreatic tumors in humans are hypoxic, with few blood vessels as evidenced by histological examination, this being partially explained by a dense desmoplastic stroma (Erkan et al., 2009). We therefore investigated the effect of hypoxia on PDAC cell supernatant polarized macrophages (in full) and compared the effect to cytokine-stimulated macrophages. Myeloid cells were co-cultured as described earlier with one of the PDAC cell lines (CFPAC) that induced the highest MARCO expression. The phenotype driven by the hypoxic conditions was investigated in parallel with the *in vitro* generated pro-inflammatory (LPS/IFNγ) and anti-inflammatory (IL-4/IL-10) macrophages. Co-cultures were incubated in normoxic or hypoxic conditions for 24 h and their phenotype and cytokine profile were assessed. We found a significant upregulation of MARCO and high production of ARG-I in hypoxia, and a trend of higher CD163 and CD206 expression, lower expression of CD86, and lower production of IL-12. No change in HLA-DR or VEGF was observed relative to normoxia (Figure 3A). In addition, TCM in hypoxia had higher expression of MARCO and ARG-than when cultured in normoxic conditions. Regardless of hypoxia, TCM displayed a low HLA-DR expression together with high ARG-I production, which is a feature of cytokine-generated (GM-CSF + IL-6) MDSC (Gabrilovich et al., 2012; Ostrand-Rosenberg and Sinha, 2009). Notably, we found that the MDSC-like anti-inflammatory macrophages expressed MARCO, although their inflammatory profile was distinct (Figure 3B). In conclusion, our data demonstrate hypoxia-enhanced MARCO expression in the immunosuppressive myeloid compartment comprising both MDSCs and TAM populations.

### Pancreatic cancer cells drive MARCO-expressing myeloid cells that inhibit T cell and NK cell activation

**Figure 4:**
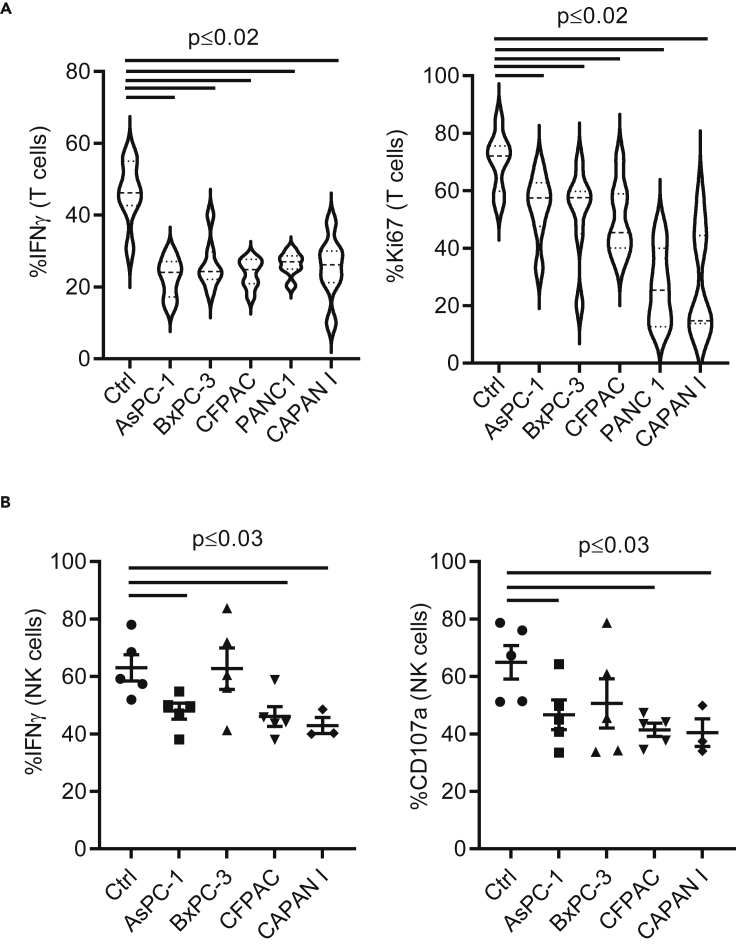
PDAC-conditioned MARCO expressing myeloid cells inhibit T and NK cell activities Myeloid cells were conditioned with PDAC cell lines for 48 h prior to co-culture with (A) T cells and (B) NK cells for 3 days at a 1:1 ratio and evaluated for IFNγ production, proliferation (Ki67), and degranulation (CD107a). T cells were assessed following CD3/CD28 activation in a mixed lymphocyte reaction (MLR) and NK cells with IL-15. NK and T cell function was then evaluated by flow cytometry following stimulation with PMA and ionomycin for 6 h prior to staining. Pooled data are presented as mean ± SEM or range and statistical analyses were performed using one-way ANOVA.

Both TAMs and MDSCs are key actors in the TME to provide immune evasion that leads to tumor progression and metastasis (Noy and Pollard, 2014; Vinay et al., 2015). *In vitro*-generated anti-inflammatory macrophages and MDSC were tested functionally and confirmed to suppress NK and T cells, and could therefore be used as suppressive myeloid cell controls (Figures S2A and S2B). Like cytokine-polarized TAMs and MDSCs, we found that tumor-cell-differentiated MARCO^+^ TCM, independent of cell-to-cell contact, could suppress cytotoxic T cell and NK cell IFNγ production, proliferation, and/or degranulation (CD107a). All tumor cells differentiated naive macrophages into TCM cells which suppressed immune effector functions relative to control macrophages cultured without tumors (Figure 4A and 4B). Our data reveal a robust immune suppression associated with the presence of MARCO^+^ TCM demonstrated by repression of activation of both NK and cytotoxic T cells.

### Targeting scavenger receptor MARCO with novel specific antibodies repolarizes human myeloid cells

**Figure 5:**
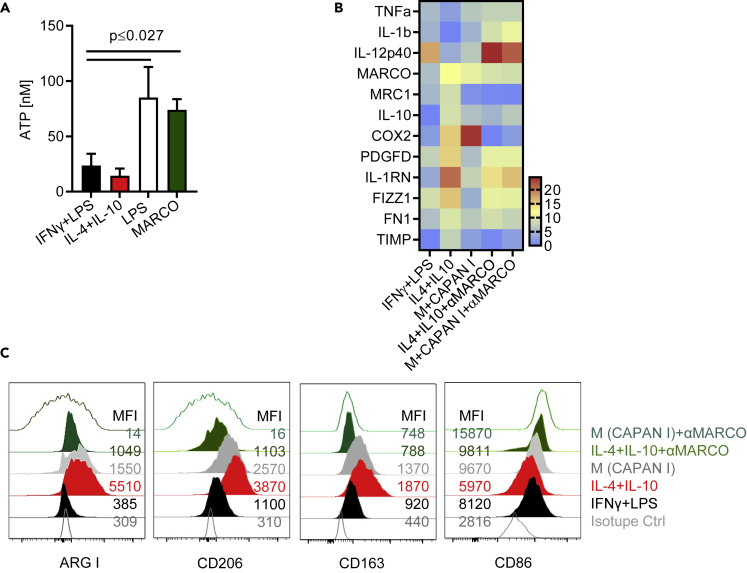
Production of anti-hMARCO antibodies and their potential effect on myeloid cells (A) Macrophages were cytokines polarized with indicated cytokines in the absence or presence of anti-hMARCO antibody clones for 10 h and extracellular ATP release was measured with a luminometer. Pooled data (n = 4–6) shown as mean ± SEM and statistical analysis were performed using Mann-Whitney U-test. (B) Relative expression of RT-qPCR for analyzing the expression of pro-inflammatory (Tnfa, Il12p40, Il1b) and anti-inflammatory markers (MARCO, Mrc1, Il10, COX2, PFGFD, Il1rn, TIMP1, FN1) in cytokine polarized macrophages or TCM in the absence or presence of anti-hMARCO antibodies. Pooled data (n = 4) were normalized to GAPDH expression and shown as a heatmap. Protein expression analyses by flow cytometry of ARG I, CD206, CD163, and CD86 in macrophages polarized as indicated in the presence of (C) anti-hMARCO antibodies. Representative histograms are shown from at least 4 independent experiments.

We previously reported that using antibodies against MARCO represents an effective immunotherapy in mice, reducing tumor growth and blocking metastasis in several cancer models. We also determined that targeting MARCO^+^ TAMs resulted in the release of NK-cell-mediated killing *in vitro* (Georgoudaki et al., 2016; La Fleur et al., 2021). This effect was accompanied by reprograming macrophages toward a pro-inflammatory phenotype. We thus next investigated whether similar effects were evident in human myeloid cells in the context of PDAC. To test this, we immunized MARCO-gene-deleted mice with recombinant human MARCO protein in order to induce the production of mouse anti-hMARCO antibodies. The antibody binding efficacy was tested using ELISA against recombinant human MARCO protein and additionally by binding analysis of human MARCO-transfected CHO cells (Eisinger et al., 2020). Furthermore, macrophage viability was assessed following anti-hMARCO treatment, with no difference compared to treatment with control IgG (Figure S3A). Several anti-MARCO-producing B cell hybridoma clones were tested, and one was selected for further studies. We have previously shown that engagement of MARCO using antibodies results in ATP release in murine macrophages (Prokopec et al., 2016). To examine whether targeting human MARCO mediated a similar effect, we assessed the ATP release of *in vitro* generated pro and anti-inflammatory macrophages in the presence or absence of the selected anti-hMARCO antibody clone compared to macrophages activated with LPS used as a positive control. We observed that the selected anti-MARCO clone induced extracellular release of ATP (Figure 5A). Based on the binding capacity and induction of ATP release data we confirmed that the anti-hMARCO clone selected induced functional changes and could thus be used for further analyses. In order to investigate whether anti-MARCO engagement could reprogram anti-inflammatory macrophages, we next analyzed changes in mRNA expression of macrophage-expressed genes in anti-hMARCO treated IL-4/IL-10 macrophages compared to LPS/IFNγ treated macrophages or TCM in the presence or absence of anti-hMARCO treatment. We found that anti-MARCO treatment reduced the expression of immunosuppressive genes associated with anti-inflammatory macrophages and TCM, including MRC1, IL-10, COX2, and TIMP1 and increased the expression of pro-inflammatory genes such as TNFα, IL-12p40, and IL-1β (Figure 5B). We also detected a reduction in protein levels of Arg I, CD206, and CD163 and an increase in the expression of the co-stimulatory molecule CD86 (Figure 5C).

### Targeting human MARCO induces T cell and NK-cell-mediated killing of human pancreatic cancer cells

**Figure 6:**
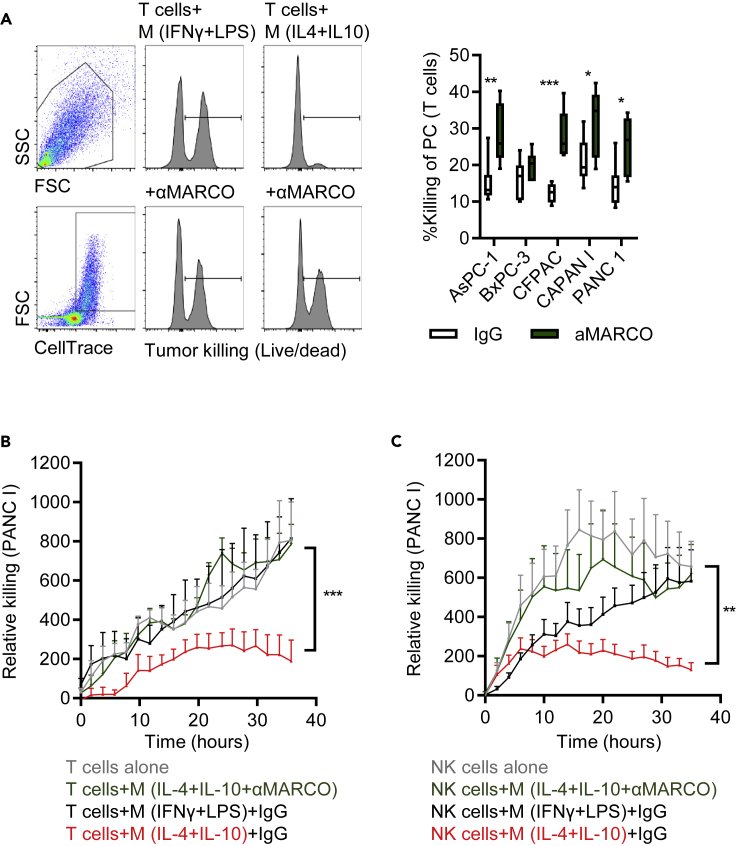
Targeting MARCO protects from myeloid immune suppression (A) T cells were co-cultured with cytokine-indicated or PDAC-conditioned myeloid cells and their PDAC killing capacity was measured. CellTrace labeled PDAC cells were added to the T cell - macrophage cultures and the percentage of dead tumor cells was measured by live/Dead marker analyzed by flow cytometry. (B) T cells or (C) NK cells were cultured alone, or co-cultured with macrophages polarized with indicated cytokines ± anti-hMARCO, for 3 days at a 1:1 ratio and then killing capacity against PANC1 was monitored by hourly fluorescence imaging over 30 h using IncuCyte Live Cell Analysis System. Pooled data (n = 4) are presented as mean + SEM and statistical analyses were performed using multiple comparison tests and p values were corrected using FDR (FDR <0.05 was considered significant). ∗∗p ≤ 0.001, ∗∗∗p ≤ 0.0001.

We tested if the newly developed anti-hMARCO antibodies could target and alter the immunosuppressive TCM and subsequently release the block of T cells and NK cells. Anti-hMARCO antibody pre-treated macrophages increased T cell cytotoxicity toward several human PDAC cell lines. Notably, TCM treated with anti-hMARCO prior to co-culture with T cells reversed the inhibition of anti-tumor activities of T cells which were now able to kill respective tumor cells (Figure 6A). The effect was dependent on macrophages as T cells cultured alone with anti-hMARCO did not induce any changes in IFNγ production (Figure S3B). Strikingly, we found that T cells and NK cells co-cultured with anti-hMARCO-treated anti-inflammatory macrophages regained their ability to kill PDAC cell lines as evidenced using long-term cytotoxicity assays (Figures 6B and 6C). Collectively, these data demonstrate that the biology of targeting MARCO in humans is similar to that in mice and support the development of treatments that promote T-cell- and NK-cell-mediated effects in PDAC.

### Activation of the inflammasome supports cellular cytotoxicity

**Figure 7:**
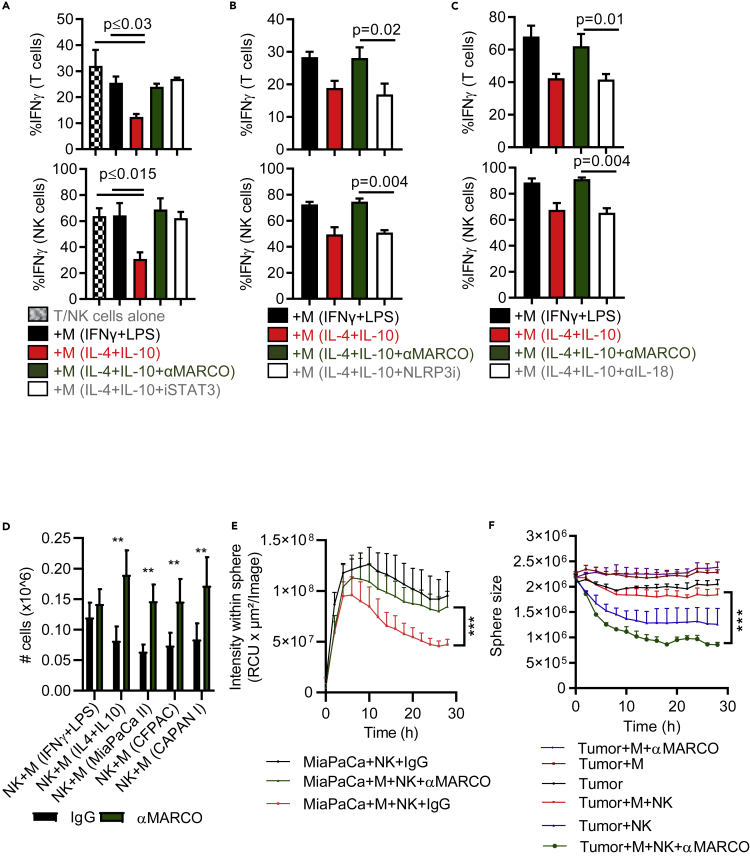
MARCO expressing myeloid cells limit T and NK cell infiltration (A–C) T cells or NK cells were cultured alone, or co-cultured with macrophages polarized with indicated cytokines ± anti-hMARCO, ± STAT3 or NLRP3 small molecule inhibitor, or anti-IL18, for 3 days at a 1:1 ratio and evaluated for IFNγ production. Pooled data are presented as mean ± SEM and statistical analyses were performed using multiple comparison tests and p values were corrected using FDR (FDR <0.05 was considered significant). (D) NK cell migration was evaluated in transwell assays allowing for only active migration of NK cells, laid onto the upper insert, toward the bottom where indicated macrophages were cultured. Pooled data (n = 5) are presented as mean ± SEM. (E and F) PDAC cell line MiaPaCa II was mixed with purified macrophages from healthy blood donors and cultured in a low attachment sphere plate for 5 days in the presence or absence of anti-hMARCO antibodies prior to culture with dye-labeled NK cells. NK cell infiltration and sphere size were assessed over time using the Incucyte-live cell imaging system. Pooled data (n = 3) are shown as mean + SEM. (D–F) Statistical analyses were performed using multiple comparison tests and p values were corrected using FDR (FDR <0.05 was considered significant). ∗∗p ≤ 0.001, ∗∗∗p ≤ 0.0001.

Given the regulatory role of STAT3 in macrophage MARCO expression we next tested the inhibition of STAT3 in macrophages prior to T and NK cell functional assays and compared this to treatment with anti-hMARCO. By inhibiting STAT3 activity we reversed macrophage inhibition of IFNγ production by T and NK cell co-cultures to similar levels as when treated with anti-MARCO (Figure 7A), suggesting that anti-hMARCO treatment can overcome high STAT3 macrophage suppression. Given that STAT3 can up-regulate HIFα (Niu et al., 2008), which we found binding to the MARCO promotor, we investigated whether HIFα expression is increased in normoxic conditions following treatment with IL-10 and whether anti-hMARCO treatment can prevent the expression of HIFα. Indeed, polarizing macrophages with IL-10 induced HIFα expression that in turn was impaired by anti-hMARCO treatment (Figure S3C). We reported earlier that anti-hMARCO treatment induces extracellular ATP release and it is known that ATP activates the NLRP3 inflammasome, which results in IL-18 and IL-1β maturation and release (Piccini et al., 2008). We thus first tested whether catalyzing ATP by ATPase would abolish anti-hMARCO treatment effects. We found that ATPase in combination with anti-hMARCO treatment reduced T cell proliferation and IFNγ production back to the same levels as before anti-hMARCO treatment, in the presence of anti-inflammatory macrophages (Figure S3D). We next investigated whether inhibition of the NLRP3 inflammasome and IL-18 influenced T and NK cell functions and could interfere with the effect of anti-hMARCO treatment. We added a specific small-molecule NLRP3 inhibitor (Coll et al., 2015) during anti-hMARCO pre-treatment and IL-18 neutralizing antibodies during co-cultures with T cells or NK cells. Blocking of either NLRP3 or IL-18 abrogated the anti-hMARCO treatment effect, resulting in persistent inhibition of T cell and NK cell IFNγ production (Figures 7B and 7C). Taken together these data indicate that anti-hMARCO treatment starts an ATP-dependent activation chain that reprograms immunosuppressive myeloid cells that leads to activation of cytotoxic lymphocytes in an IL-18-dependent manner.

### Anti-hMARCO treatment of myeloid cells induces NK cell tumor infiltration and restores their killing capacity *invitro*

We have demonstrated that MARCO^+^ macrophage presence in the TME of PDAC is associated with a lymphocyte-excluding tumor phenotype. To further investigate the underlying mechanisms, we examined NK cell migration toward PDAC lines cultured with macrophages using a 2D transwell migration assay. NK cell infiltration into 3D pancreatic tumor spheroids cultured with macrophages in long-term live cell imaging was also investigated. In the 2D cultures we observed less migration of NK cells toward anti-inflammatory macrophages or to tumor cells cultured with macrophages, compared with migration toward pro-inflammatory macrophages (Figure 7D). In the 3D model there was a decreased infiltration of NK cells into tumor spheres formed in the presence of macrophages compared with tumor spheres in the absence of macrophages (Figures 7E and S4A). This effect was not due to macrophage depletion as the macrophages were equally viable following anti-hMARCO treatment in the 3D model (Figure S4B). Furthermore, poor NK cell infiltration correlated with impaired tumor targeting, as represented by increased sphere size in the 3D PDAC model (Figure 7F). Targeting MARCO in these cultures markedly increased NK cell migration toward 2D tumor cells and into 3D tumor spheroids and improved anti-tumor activity.

## Discussion

Using *in vitro* generated and tumor-conditioned suppressive myeloid cells, we demonstrate in this study that expression of the scavenger receptor MARCO is associated with an immunosuppressive microenvironment characterized by lymphocyte exclusion, tumor progression and poor survival in patients with pancreatic cancer. We show that pancreatic cancer cell conditioning of myeloid cells induces suppressive MDSC/TAM like phenotypes and functions. These suppressive myeloid cells expressed high levels of MARCO activated by tumor derived IL-10 that was induced via the transcription factor STAT3. Furthermore, we show that targeting human MARCO with antibodies repolarizes myeloid cells toward a pro-inflammatory phenotype, inhibiting their suppressive capacity on T cells and NK cells through mechanisms involving extracellular ATP release, inflammasome activation and IL-18 production.

Current treatments targeting pancreatic cancer are inefficient, partly due to a dense stroma comprising up to 80% of the tumor that builds a complex immunosuppressive environment (Shakya et al., 2013). Conversely, immunotherapy has lately gained attention and has become a well-established anti-cancer therapy (Dougan and Dranoff, 2009). However, less than 50% of all cancer patients are expected to respond to the current immune checkpoint therapies, even in the cancers in which it is most effective. New targets for immunotherapy are therefore highly desirable and especially those that increase the efficacy of T cell targeting (Sharma and Allison, 2015). TAMs and MDSCs within the TME control tumor development and shape the anti-tumor responses, affecting the clinical outcome. We previously identified a subpopulation of MARCO- expressing macrophages highly represented in the TME in human breast cancer, metastatic melanoma, periampullary adenocarcinoma (intestinal type), and small cell lung cancer (Georgoudaki et al., 2016; La Fleur et al., 2018; Lundgren et al., 2017). We now report a high prevalence of MARCO-expressing macrophages in PDAC. Targeting MARCO with a monoclonal antibody in murine breast and colon cancer models reprograms TAMs toward a pro-inflammatory phenotype (Georgoudaki et al., 2016). In the current study, we confirm the potential of targeting human MARCO as a next-generation immunotherapy in pancreatic cancer. MARCO is normally expressed by subpopulations of macrophages where it works as a pattern recognition receptor by binding both foreign and self-ligands. The receptor has no signaling domain, and so far, no co-receptor has been defined. Thus, the signaling pathway triggered by anti-hMARCO antibodies remains to be defined. Data from murine macrophages show fast internalization and both in humans and in mice there is a release of extracellular ATP.

The host immune system plays a key role within the TME whereby different immune cells have contrasting impacts on pancreatic cancer progression; while high infiltration of CD8^+^ T cells is a positive prognostic factor, macrophage and MDSC infiltration is negatively correlated with survival (Ene-Obong et al., 2013; Ino et al., 2013). We have previously described in human adenocarcinoma that extensive infiltration of MARCO^+^ TAMs is associated with resistance to chemotherapy (Lundgren et al., 2017). Herein we report that a high prevalence of MARCO^+^ myeloid cells in the tumor area is associated with exclusion of NK and T cells in PDAC. Exclusion of T cells in PDAC has been highlighted as a resistance mechanism for checkpoint immunotherapies using anti-PD1 and anti-CTLA-4 (Joyce and Fearon, 2015). Peranzoni et al. provided evidence of involvement of TAMs as a key player in this lymphocyte-excluded tumor phenotype (Peranzoni et al., 2018). Based on these studies and our current results we hypothesize that reprogramming the immunosuppressive and lymphocyte-excluding myeloid cells through anti-MARCO treatment can be a potential approach to re-recruit immune effector cells to the tumor area and convert cold tumors into hot tumors.

As we observed a generally high MARCO expression in pancreatic cancer and since its expression was connected to survival, this indicates its usefulness for targeting. The fact that MARCO is only normally expressed by a very limited number of macrophages makes it potentially a very specific intervention. Herein we prove that several pancreatic cancer cell lines induce myeloid suppressor cell phenotypes through production of IL-10. The effect included lower expression of HLA-DR (MHC-II) and induction of a phenotype resembling TAMs and MDSCs (Cassetta and Pollard, 2018; Marvel and Gabrilovich, 2015). Besides tumor cells, other immune cells are also IL-10 producers in the TME, including MDSC, TAMs, and regulatory T cells, that contribute to an autocrine- and paracrine-induced immunosuppressive environment. Targeting IL-10 in the TME has been shown in translational studies to enhance T cell anti-tumor responses in otherwise immune cold tumors lacking the infiltration of T cells (Teng et al., 2011). The targeting of IL-10 might indirectly remodel the suppressive myeloid compartment to inhibit their mediated immune exclusion. Conversely, systematic inhibition of IL-10 can mediate adverse effects in the management of inflammatory diseases in which IL-10 plays a significant role (Couper et al., 2008). The specific targeting of suppressive myeloid cells in the TME is thus needed, and this could be achieved through targeting MARCO.

Two characteristics of pancreatic cancer, hypoperfusion and desmoplasia, play leading roles in the formation of a hypoxic microenvironment. The hypoxia inducible factor transcription family (HIF) helps cells to adapt to a hypoxic environment (Chang et al., 2011). HIF-1 is highly expressed in 88% of pancreatic cancer tissues and its overexpression is correlated with poor prognosis (Erickson et al., 2015). HIF-1α is known to promote the differentiation of MDSCs and TAMs and their immunosuppressive activities (Kumar and Gabrilovich, 2014). The question remained whether hypoxia also has an effect on MARCO expression in MDSCs and TAMs, and our data revealed an increased expression. Overall, hypoxia amplified the expression of pro-tumoral macrophage markers and de-differentiated myeloid cells toward MDSCs. In concordance with our findings, Corzo et al. demonstrated that hypoxia is a key player in MDSC differentiation and function via HIF-1α (Corzo et al., 2010). Our data indicate that the hypoxic microenvironment induces a prominent immunosuppressive myeloid subtype. Future functional assays using 3D tumor spheroids and myeloid cell culture under hypoxic conditions will serve to elucidate the effect of anti-hMARCO treatment on the TME organization.

Our examination of human tumors revealed that T cells and NK cells appeared to be absent in areas positive for MARCO, suggesting that these cells could potentially block entry and activation/migration in the tumor. Here, we showed that cytokine-induced anti-inflammatory and PDAC-induced myeloid cells have the ability to inhibit T cell and NK-cell proliferation, as well as NK cell migration. In addition, they lead to reduced IFNγ-production and cytolytic activity involving ATP-dependent IL-18 activation pathways. Previous studies have emphasized the role of IL-18 in augmenting anti-CTLA-4 and anti-PD-L1 checkpoint immunotherapies through increased survival and expansion of cytotoxic CD8^+^ T cells and accumulation of mature NK cells (Ma et al., 2016; Souza-Fonseca-Guimaraes and Huntington, 2018). Anti-hMARCO treatment could thus be used as a combinatory immunotherapy with anti-CTLA-4/anti-PD-1/PD-L1 to improve the efficacy of checkpoint therapies in pancreatic cancer patients.

### Limitations of the study

The *in vitro* polarization was performed with an anti-hMARCO antibody that was of mouse origin. Thus, it will be of importance to humanize anti-hMARCO antibodies to test the involvement of Fc-receptors that differ between humans and mice. Also *in vivo* studies using hMARCO expressing mice with hFcR will be needed to test a humanized potential clinical product. The cohort of patients studied here is limited and aimed to show that MARCO is expressed and can be used as a target. A larger cohort should be studied to evaluate the usage of MARCO staining as a marker for treatment outcomes etc.

## STAR★Methods

### Key resources table

**Table:** 

REAGENT or RESOURCES	SOURCE	IDENTIFIER
**Antibodies**		

CD107a	Biolegend	H4A3 Cat#328616
CD56	Biolegend	5.1H11 Cat#362510
Ki67	BD	B56 Cat# 561277
IFNg	BD	4S.B3 Cat# 502538
CD3	BD	OKT3 Cat#566781
CD206	Biolegend	19.2 Cat#551136
HLADR	Biolegend	L243 Cat# 361608
CD86	BD	2331 (FUN-1) Cat# 344712
CD68	BD	Y1/82A Cat# 564943
CD14	Biolegend	63D3 Cat# 367140
CD163	Biolegend	GH1/61 Cat# 333628
IL-10	Biolegend	JES3-19F1 Cat# 501404
TNFα	Biolegend	MAb11 Cat# 502940
IL-12	BD	C8.6 Cat# 565023
ARG I	Biolegend	14D2C43 Cat# 369704
VEGF	R&D	23,410 Cat# IC2931A
CD68	Abcam	KP1 Cat#Ab955
CD3	Bio-Rad	CD3-12 Cat#MCA1477
CD56	Invitrogen	3HCLC Cat#710388
STAT3 Recombinant Polyclonal Antibody	ThermoFisher Scientific	Cat #710077

**Chemicals, peptides, and recombinant proteins**		

Dead cell marker	ThermoFisher Scientific	Cat#L34976
RPMI medium	ThermoFisher Scientific	Cat #22-400-089
FBS	Gibco	Cat #A4766801
Penicillin/Streptomycin	Merck Life Science	Cat# P4333
LPS	InvivoGen	Cat# tlrl-eblps
hIFNg	Peprotech	Cat #300-02
hM-CSF	Peprotech	Cat #300-25
Dynabeads™ Human T-Activator CD3/CD28	ThermoFisher Scientific	Cat #11131D
NLRP3 inhibitor MCC950	Sigma Aldrich	Cat #5381200001
ADENOSINE 5′-TRIPHOSPHATASE ATPase	Sigma Aldrich	Cat #A7510-5UN
CellTrace Violet	ThermoFisher Scientific	Cat #C34557
CellTracker deep red	ThermoFisher Scientific	
Caspase3/7 green dye	ThermoFisher Scientific	Cat #C10423
SYBR Green	Bio-Rad	N/A

**Oligonucleotides**		**Cat #C34565**

Primer for qPCR in Table S2	Table S2	Sigma Aldrich costumed
Primers for ChIP assay in Table S2	Table S2	Merck

**Experimental Models: Cell lines**	Table S1	**N/A**

**Biological Samples**		

Human Buffy coats	Stockholm Blood Bank	N/A
Pancreatic tumor sections	Pathology Division Karolinska University Hospital	

**Critical commercial assays**		

RNeasy mini Kit	Qiagen	Cat #74004
NK cell isolation Kit	Miltenyi Biotec	Cat #130-092-657
Pan Monocyte Isolation Kit, human	Miltenyi Biotec	Cat #130-096-537
CD3 microbeads	Miltenyi Biotec	Cat #130-050-101
MARCO sandwich ELISA	Mabtech AB	Custom
ClonaCell™-HY Hybridoma Kit	STEMCELL Technologies, Inc.	Cat #03800
ATP Determination Kit	ThermoFisher Scientific	Cat #A22066
Pierce™ Chromogenic Endotoxin Quant Kit	ThermoFisher Scientific	Cat #A39553
Millipore ChIP assay kit	Merck	Cat #17-296

**Software and algorithms**		

GraphPad Prism v8.01	GraphPad Software	https://www.graphpad.com/
FlowJo 10.8	Treestar	https://www.flowjo.com/solutions/flowjo
BD FACSDiva 8.0.2	BD Bioscience	N/A
LSM800 confocal microscope	Zeiss	N/A
IncuCyte Live Cell Analysis System	Essen BioScience	N/A
IncuCyte S4 software	Essen BioScience	N/A
ImageJ software	NIH	https://imagej.nih.gov/ij/
R software v3.6.1	R	https://www.r-project.org/
PDAC cohort	The Cancer Genome Atlas (TCGA)	http://gepia.cancer-pku.cn
Normal tissues	Geno-type-Tissue Expression (GTEx)	https://doi.org/10.1093/nar/gkz430
GSE28735	Gene Expression Omnibus – NCBI (GEO)	https://www.ncbi.nlm.nih.gov/search/all/?term=GSE28735

### Resource availability

#### Lead contact

The Study materials will be provided after a reasonable request. Inquiries can be directed to the lead contact, Dr. Mikael C.I. Karlsson.

#### Materials availability

Inquiries can be directed to the lead contact, Dr. Mikael C.I. Karlsson.

### Experimental model and subject details

#### Patients and healthy donors

Pancreatic tumor collection was approved by the regional ethics review board in Stockholm (Dnr. 2013.977-31.1). Informed consent was obtained from all subjects. Human biological samples consisting of tumor tissue and healthy donor (HD) blood, were sourced ethically and their research use was in accordance with the terms of the informed consents under the approved protocol. Cryopreserved peripheral blood mononuclear cells (PBMC) from HD blood donors, were obtained following Ficoll-Hypaque density gradient purification. Blood from HD were procured from the Stockholm Blood Bank. Donors’ information was deidentified before receiving.

#### Human tissue expression profiling

GEPIA (http://gepia.cancer-pku.cn), a freely available comprehensive web-based tool was used to analyze expression data at the transcriptional level from The Cancer Genome Atlas (TCGA) and The Geno-type-Tissue Expression (GTEx) projects (Tang et al., 2019). GEPIA was also used to analyze the mRNA expression level of MARCO in PDAC. The normalized RNA sequencing data (transcripts per million, TPM) and corresponding clinical and pathological data of PDAC were obtained from TCGA (https://portal.gdc.cancer.gov/) up to March, 20th, 2020. A total of 150 PDAC patients were selected from 185 all pancreatic cancer patients in the TCGA cohort (The gender/sex influence was not investigated in this study, thus no gender/sex information is provided or not available). SurvivalROC package was used to calculate the best cut-off value (16.10, min 9.45, max 21.64) and data were divided into MARCO high cohort (n = 112) and MARCO low cohort (n = 38). Statistical analyses were performed in R software v3.6.1 (https://www.r-project.org/) and Kaplan-Meier survival curve displayed in GraphPad Prism v8.01 (https://www.graphpad.com/).

### Method details

#### Histology, immunohistochemistry, and microscopy

For histological analysis, pancreatic specimens were fixed with formalin, dehydrated in ethanol, embedded with paraffin, sectioned and stained with H&E. Immunofluorescent staining on human paraffin-embedded or frozen tissues was performed using antibodies directed against CD68, MARCO, CD3, alternatively CD56 and Hoechst. For paraffin-embedded slides, samples were pre-heated at 65°C for 2 h before deparaffinization in xylene and ethanol followed by antigen retrieval with a citrate-based antigen unmasking solution (Vector Labs). Immunofluorescent images were acquired using a Zeiss LSM800 confocal microscope and analyzed by ImageJ. Definition of the tumor areas was assisted by the H&E staining as well as by tumor cell morphology in IF images. MARCO + areas were defined by clusters of double-positive CD68 and MARCO stained cells.

#### Macrophage isolation and polarization

Monocytes were isolated from HD PBMC and cultured for 6 days in M-CSF and overnight polarized toward pro-inflammatory or anti-inflammatory macrophages with LPS (200 ng/mL)+IFNγ (20 ng/mL) or IL-4 (20 ng/mL)+IL-10 (20 ng/mL) respectively (Figure S5A). Fresh medium supplemented with M-CSF was added at day 3. Alternatively, differentiated macrophages at day 6 were overnight polarized with different cytokine (20 ng/mL) combinations for MARCO expression analysis including; IL-10, IL-4, IL-13, TGFβ. Further, *in vitro* MDSC were generated by treating monocytes with IL-6 (10 ng/mL) and G-MCSF (10 ng/mL) for 7 days and refreshed on day 3 or 4 with cytokine supplemented medium. All recombinant cytokines were purchased from PeproTech.

#### **Tumor-**conditioned **macrophages under normoxic and hypoxic conditions**

M-CSF differentiated macrophages were overnight polarized with either LPS + IFNγ, IL-4+IL-10, or cultured with PDAC cell lines (authenticated from ATCC, used within three months of the first passage, Table S1) (Figure S5B) referred to as tumor conditioned macrophages (TCM), separated by transwell inserts allowing for only soluble factor exchange, under normoxic (21% O_2_) or hypoxic (1% O_2_, hypoxia chamber) conditions. Subsequently, cells were examined with flow cytometry for phenotype and function, or co-cultured with effector cells to assess their immune suppressive capacity.

#### Quantitative RT-PCR

For quantification of gene expression, RNA was isolated from macrophages by RNeasy mini Kit (Qiagen). cDNA was synthesized from RNA using Superscript IV Reverse transcription (ThermoFisher) (37°C for 15 min, 65°C for 10 min). qRT-PCR reactions were performed using SYBR Green Master Mix (Applied Biosystems). Primers used for analysis of gene expression and mRNA quantification were for human TNFa, IL-1b, IL-12p40, MARCO, MRC1, IL-10, COX2, PDGFD, IL-1RN, FIZZ1, FN1, MMP12, TIMP and GAPDH (Sigma Aldrich, Table S2). All reactions were carried out using a Bio-Rad thermal cycler, Applied Biosystems 7500 Real-Time PCR System.

#### NK and T cell function assays

CD56^+^CD3^−^ NK cells were isolated using a negative depletion kit (Miltenyi). T cells were isolated using CD3 positive selecting microbeads (Miltenyi). NK cells were co-cultured with macrophages or MDSC at a 1:1 ratio in the presence of IL-15 (10 ng/mL) and evaluated for degranulation, IFNγ production, and proliferation following 5 days of co-culture. T cell proliferation and IFNγ production were assessed following stimulation using CD3/CD28 activation in a mixed lymphocyte reaction (MLR) for 3 days. Occasionally, macrophages were treated with anti- MARCO antibodies (made in house) in the presence or absence of the inflammasome NLRP3 inhibitor MCC950 (10 μM, Sigma Aldrich) or STAT3 small molecule inhibitor (Stattic, 10 μM) (Schust et al., 2006) kindly provided by the Grander research group at Karolinska Institutet, and washed prior to co-culture with cytotoxic cells. Alternatively, anti-hMARCO treatment in the presence or absence of ATPase (0.1 IU, Sigma Aldrich) anti-IL-18 (2 μg/mL, R&D Systems) antibodies in co-cultures of cytotoxic cells and macrophages. NK and T cell function was then evaluated following stimulation with PMA (100 ng/mL) and ionomycin (500 ng/mL) for 6 h prior to staining.

#### Flow cytometry analysis

For phenotypic analyses, cytokine-generated macrophages and MDSC, monocytes, and TCM were examined for the surface marker expression of CD14, HLADR, CD68, CD86, CD163, CD206 (key resources table). Subsequently, tumor cell lines, macrophages, MDSC, NK cell and T cell intracellular protein production was assessed by intracellular staining, following fixation and permeabilization (eBioscience) according to the manufacturer’s instructions, using fluorochrome-conjugated antibodies against IL-10, TNFα, IL-12, ARGI, VEGF, IFNγ, Ki67 (proliferation), and CD107a (degranulation) (key resources table). All cells were acquired by LSRII and analyzed by FlowJo 10.0.

#### Flow cytometry-based killing assay

T were added to CellTrace Violet (5 uM, Invitrogen) fluorescently labeled tumor cells, and target killing was evaluated using Live/Dead dye (Invitrogen) following a 6 h incubation at an effector to target (E:T) ratio of 3:1. Tumor cell killing was assessed by gating on the CellTrace positive population representing tumor cells and assessed for the proportion of Live/Dead positive cells (Figure S5C).

#### Live kinetic analysis of tumor cell killing

For analysis of tumor cell killing one representative pancreatic cell line PANC1 was labeled with red fluorescent CellTracker (5 uM, ThermoFisher Scientific) and plated at a concentration of 2×10^4^ cells per well in 96-well flat bottom plates. Prior to analysis, NK cells or T cells were added at a 2:1 ratio onto the target cells and caspase3/7 dye (green, ThermoFisher Scientific) was added to all wells. The number of killed target cells was monitored by hourly fluorescence imaging over 36 h using an IncuCyte Live Cell Analysis System (Essen BioScience) (Figure S5D). Relative killing (green/red overlap) was quantified using IncuCyte S4 software (Essen BioScience) and normalized to the number of cells in each well at the start and number of spontaneous cell death in the target cells only control group.

#### Anti-hMARCO antibody production

Anti-hMARCO antibodies were produced as described earlier (Eisinger et al., 2020). Briefly, 3 female C57BL/6 MARCO knock-out mice at 8 weeks of age were immunized with 50 ug human MARCO recombinant protein in 100 L PBS+100 μL Gerbu adjuvant intraperitoneal injected. Mice were boosted twice before collection of the spleens. anti-hMARCO antibody production was performed according to the manufacture procedure (ClonaCell-HY Hybridoma Cloning kit, Stemcell Technologies). In brief, splenocytes were fused with myeloma cells at 5:1 ratio. After colony formation, individual colonies were disrupted in individual wells in 96-well plates and later tested for MARCO specificity in a sandwich ELISA (Mabtech AB). Specific clones were expanded, and supernatant was collected for purification of antibodies. Culture supernatants were collected, and the antibodies were isolated by standard protein purification techniques using G-protein-specific separation columns.

#### Extracellular ATP detection

Macrophages were polarized overnight with LPS + IFNγ, IL-4+IL-10, or IL-4+IL-10+anti-hMARCO antibody (10 μg/mL). Following 10 h culture, medium was collected and assessed for extracellular ATP following the manufacture procedure (Molecular ProbesTM, Thermofisher Scientific).

#### Endotoxin and isotype test

Following purification of anti-hMARCO antibody, endotoxin quantification was performed using the Pierce LAL Chromogenic Endotoxin Quantitation Kit via a chromogenic signal generated in the presence of endotoxins (ThermoFisher Scientific). Later, we assessed our in-house produced mouse anti-hMARCO isotype by ELISA.

#### ChIP

Monocytes (12M) were isolated from HD PBMC and cultured for 5 days in M-CSF and overnight polarized toward anti-inflammatory macrophages with IL-4 (20 ng/mL)+IL-10 (20 ng/mL). Chromatin Immunoprecipitation (n = 3) was performed using Millipore ChIP assay kit (17–296, Merck) according to the manufacturers' instructions. Chromatin was sheered by sonication (Bioruptur, Diagenode), 30 s on/30 s off, for 12 min. Antibodies against human (h)STAT3 (Ref: 710,077, Thermo Fisher Scientific) or against IgG (negative control) were used. Detection of ChIP signal was done by qPCR (Rotor Gene RG-3000A, Corbett) using SYBR Green (Bio-Rad). Fold enrichment of amplified regions were calculated as Ct_IgG_-Ct_STAT3_. Primers used for detection included in Table S2.

#### 3D Co-cultures and NK cell infiltration

3D co-cultures were performed in low-attachment 96 well U-bottom plates (Nunclon Sphera 96 Well microplate, ThermoFisher Scientific). 0.8-1x10^4^ MiaPaCa-2 cells were seeded per well alone as monoculture or together with 1.6-2x10^4^ human primary monocytes as co-culture and M-CSF (100 ng/mL) was added on day 0 and day 3 to induce macrophage differentiation. Monocultures and co-cultures were incubated in the presence or absence of α-MARCO (10 μg/mL) for 5 days at 37 and 5% CO_2_ until spheroid formation. On day 5, 2 × 10^4^ autologous NK cells labeled with CellTracker Deep Red (Thermo Fisher) were added to each well. Spheroid size and NK cell infiltration were assessed with Incucyte S4 Live-cell analysis system (Sartorius) for at least 30h (Figure S5E).

### Quantification and statistical analysis

All data were first analyzed in the software mentioned above and summarized by Prism Version 8 software (GraphPad). All data were first tested for normal distribution. Thereafter, statistical differences among groups were analyzed by parametric or nonparametric T-test or multiple comparison tests and p values were corrected using FDR for multiple comparisons (FDR <0.05 was considered significant) as indicated in the figure legends. Representative histograms or images were chosen based on the average values.

## Data Availability

• Data generated in this study from the RNA sequencing data are available from the original references. • No code data was generated. • Any additional information required to reanalyze the data reported in this paper is available from the lead contact (MCIK) upon request.

## References

[bib1] Brahmer J.R., Tykodi S.S., Chow L.Q., Hwu W.J., Topalian S.L., Hwu P., Drake C.G., Camacho L.H., Kauh J., Odunsi K. (2012). Safety and activity of anti-PD-L1 antibody in patients with advanced cancer. N. Engl. J. Med..

[bib2] Cassetta L., Pollard J.W. (2018). Targeting macrophages: therapeutic approaches in cancer. Nat. Rev. Drug Discov..

[bib3] Chang Q., Jurisica I., Do T., Hedley D.W. (2011). Hypoxia predicts aggressive growth and spontaneous metastasis formation from orthotopically grown primary xenografts of human pancreatic cancer. Cancer Res..

[bib4] Clark C.E., Hingorani S.R., Mick R., Combs C., Tuveson D.A., Vonderheide R.H. (2007). Dynamics of the immune reaction to pancreatic cancer from inception to invasion. Cancer Res..

[bib5] Coll R.C., Robertson A.A.B., Chae J.J., Higgins S.C., Muñoz-Planillo R., Inserra M.C., Vetter I., Dungan L.S., Monks B.G., Stutz A. (2015). A small-molecule inhibitor of the NLRP3 inflammasome for the treatment of inflammatory diseases. Nat. Med..

[bib6] Conroy T., Desseigne F., Ychou M., Bouché O., Guimbaud R., Bécouarn Y., Adenis A., Raoul J.L., Gourgou-Bourgade S., de la Fouchardière C. (2011). FOLFIRINOX versus gemcitabine for metastatic pancreatic cancer. N. Engl. J. Med..

[bib7] Corzo C.A., Condamine T., Lu L., Cotter M.J., Youn J.I., Cheng P., Cho H.I., Celis E., Quiceno D.G., Padhya T. (2010). HIF-1alpha regulates function and differentiation of myeloid-derived suppressor cells in the tumor microenvironment. J. Exp. Med..

[bib8] Couper K.N., Blount D.G., Riley E.M. (2008). IL-10: the master regulator of immunity to infection. J. Immunol..

[bib9] Dougan M., Dranoff G. (2009). Immune therapy for cancer. Annu. Rev. Immunol..

[bib10] Eisinger S., Sarhan D., Boura V.F., Ibarlucea-Benitez I., Tyystjärvi S., Oliynyk G., Arsenian-Henriksson M., Lane D., Wikström S.L., Kiessling R. (2020). Targeting a scavenger receptor on tumor-associated macrophages activates tumor cell killing by natural killer cells. Proc. Natl. Acad. Sci. USA.

[bib11] Ene-Obong A., Clear A.J., Watt J., Wang J., Fatah R., Riches J.C., Marshall J.F., Chin-Aleong J., Chelala C., Gribben J.G. (2013). Activated pancreatic stellate cells sequester CD8(+) T cells to reduce their infiltration of the juxtatumoral compartment of pancreatic ductal adenocarcinoma. Gastroenterology.

[bib12] Erickson L.A., Highsmith W.E., Fei P., Zhang J. (2015). Targeting the hypoxia pathway to treat pancreatic cancer. Drug Des. Devel. Ther..

[bib13] Erkan M., Reiser-Erkan C., Michalski C.W., Kleeff J. (2010). Tumor microenvironment and progression of pancreatic cancer. Exp. Oncol..

[bib14] Erkan M., Reiser-Erkan C., Michalski C.W., Deucker S., Sauliunaite D., Streit S., Esposito I., Friess H., Kleeff J. (2009). Cancer-stellate cell interactions perpetuate the hypoxia-fibrosis cycle in pancreatic ductal adenocarcinoma. Neoplasia.

[bib15] Faas M.M., Sáez T., de Vos P. (2017). Extracellular ATP and adenosine: the Yin and Yang in immune responses?. Mol. Aspects Med..

[bib16] Gabrilovich D.I., Ostrand-Rosenberg S., Bronte V. (2012). Coordinated regulation of myeloid cells by tumours. Nat. Rev. Immunol..

[bib17] Georgoudaki A.M., Prokopec K.E., Boura V.F., Hellqvist E., Sohn S., Östling J., Dahan R., Harris R.A., Rantalainen M., Klevebring D. (2016). Reprogramming tumor-associated macrophages by antibody targeting inhibits cancer progression and metastasis. Cell Rep..

[bib18] Gombault A., Baron L., Couillin I. (2012). ATP release and purinergic signaling in NLRP3 inflammasome activation. Front. Immunol..

[bib19] Henze A.T., Mazzone M. (2016). The impact of hypoxia on tumor-associated macrophages. J. Clin. Invest..

[bib20] Ino Y., Yamazaki-Itoh R., Shimada K., Iwasaki M., Kosuge T., Kanai Y., Hiraoka N. (2013). Immune cell infiltration as an indicator of the immune microenvironment of pancreatic cancer. Br. J. Cancer.

[bib21] Johnson B.A., Yarchoan M., Lee V., Laheru D.A., Jaffee E.M. (2017). Strategies for increasing pancreatic tumor immunogenicity. Clin. Cancer Res..

[bib22] Joyce J.A., Fearon D.T. (2015). T cell exclusion, immune privilege, and the tumor microenvironment. Science.

[bib23] Kiss M., Van Gassen S., Movahedi K., Saeys Y., Laoui D. (2018). Myeloid cell heterogeneity in cancer: not a single cell alike. Cell. Immunol..

[bib24] Kumar V., Gabrilovich D.I. (2014). Hypoxia-inducible factors in regulation of immune responses in tumour microenvironment. Immunology.

[bib25] La Fleur L., Botling J., He F., Pelicano C., Zhou C., He C., Palano G., Mezheyeuski A., Micke P., Ravetch J.V. (2021). Targeting MARCO and IL37R on immunosuppressive macrophages in lung cancer blocks regulatory T cells and supports cytotoxic lymphocyte function. Cancer Res..

[bib26] La Fleur L., Boura V.F., Alexeyenko A., Berglund A., Pontén V., Mattsson J.S.M., Djureinovic D., Persson J., Brunnström H., Isaksson J. (2018). Expression of scavenger receptor MARCO defines a targetable tumor-associated macrophage subset in non-small cell lung cancer. Int. J. Cancer.

[bib27] Lundgren S., Karnevi E., Elebro J., Nodin B., Karlsson M.C.I., Eberhard J., Leandersson K., Jirström K. (2017). The clinical importance of tumour-infiltrating macrophages and dendritic cells in periampullary adenocarcinoma differs by morphological subtype. J. Transl. Med..

[bib28] Ma Z., Li W., Yoshiya S., Xu Y., Hata M., El-Darawish Y., Markova T., Yamanishi K., Yamanishi H., Tahara H. (2016). Augmentation of immune checkpoint cancer immunotherapy with IL18. Clin. Cancer Res..

[bib29] Mantovani A., Schioppa T., Porta C., Allavena P., Sica A. (2006). Role of tumor-associated macrophages in tumor progression and invasion. Cancer Metastasis Rev..

[bib30] Mantovani A., Sica A., Allavena P., Garlanda C., Locati M. (2009). Tumor-associated macrophages and the related myeloid-derived suppressor cells as a paradigm of the diversity of macrophage activation. Hum. Immunol..

[bib31] Marvel D., Gabrilovich D.I. (2015). Myeloid-derived suppressor cells in the tumor microenvironment: expect the unexpected. J. Clin. Invest..

[bib32] Murray P.J. (2005). The primary mechanism of the IL-10-regulated anti inflammatory response is to selectively inhibit transcription. Proc. Natl. Acad. Sci. USA.

[bib33] Neesse A., Michl P., Frese K.K., Feig C., Cook N., Jacobetz M.A., Lolkema M.P., Buchholz M., Olive K.P., Gress T.M., Tuveson D.A. (2011). Stromal biology and therapy in pancreatic cancer. Gut.

[bib34] Niu G., Briggs J., Deng J., Ma Y., Lee H., Kortylewski M., Kujawski M., Kay H., Cress W.D., Jove R., Yu H. (2008). Signal transducer and activator of transcription 3 is required for hypoxia-inducible factor-1alpha RNA expression in both tumor cells and tumor-associated myeloid cells. Mol. Cancer Res..

[bib35] Noy R., Pollard J.W. (2014). Tumor-associated macrophages: from mechanisms to therapy. Immunity.

[bib36] O'Reilly E.M., Oh D.Y., Dhani N., Renouf D.J., Lee M.A., Sun W., Fisher G., Hezel A., Chang S.C., Vlahovic G. (2019). Durvalumab with or without tremelimumab for patients with metastatic pancreatic ductal adenocarcinoma: a phase 2 randomized clinical trial. JAMA Oncol..

[bib37] O'Reilly E.M., Oh D.Y., Dhani N., Renouf D.J., Lee M.A., Sun W., Fisher G., Hezel A., Chang S.C., Vlahovic G. (2019). Durvalumab with or without tremelimumab for patients with metastatic pancreatic ductal adenocarcinoma: a phase 2 randomized clinical trial. JAMA Oncol..

[bib38] Ostrand-Rosenberg S., Sinha P. (2009). Myeloid-derived suppressor cells: linking inflammation and cancer. J. Immunol..

[bib39] Peranzoni E., Lemoine J., Vimeux L., Feuillet V., Barrin S., Kantari-Mimoun C., Bercovici N., Guérin M., Biton J., Ouakrim H. (2018). Macrophages impede CD8 T cells from reaching tumor cells and limit the efficacy of anti-PD-1 treatment. Proc. Natl. Acad. Sci. USA.

[bib40] Piccini A., Carta S., Tassi S., Lasiglié D., Fossati G., Rubartelli A. (2008). ATP is released by monocytes stimulated with pathogen-sensing receptor ligands and induces IL-1beta and IL-18 secretion in an autocrine way. Proc. Natl. Acad. Sci. USA.

[bib41] Prokopec K.E., Georgoudaki A.M., Sohn S., Wermeling F., Grönlund H., Lindh E., Carroll M.C., Karlsson M.C.I. (2016). Cutting edge: marginal zone macrophages regulate antigen transport by B cells to the follicle in the spleen via CD21. J. Immunol..

[bib42] Royal R.E., Levy C., Turner K., Mathur A., Hughes M., Kammula U.S., Sherry R.M., Topalian S.L., Yang J.C., Lowy I., Rosenberg S.A. (2010). Phase 2 trial of single agent Ipilimumab (anti-CTLA-4) for locally advanced or metastatic pancreatic adenocarcinoma. J. Immunother..

[bib43] Schmiechen Z.C., Stromnes I.M. (2020). Mechanisms governing immunotherapy resistance in pancreatic ductal adenocarcinoma. Front. Immunol..

[bib44] Schust J., Sperl B., Hollis A., Mayer T.U., Berg T. (2006). Stattic: a small-molecule inhibitor of STAT3 activation and dimerization. Chem. Biol..

[bib45] Shakya R., Gonda T., Quante M., Salas M., Kim S., Brooks J., Hirsch S., Davies J., Cullo A., Olive K. (2013). Hypomethylating therapy in an aggressive stroma-rich model of pancreatic carcinoma. Cancer Res..

[bib46] Sharma P., Allison J.P. (2015). Immune checkpoint targeting in cancer therapy: toward combination strategies with curative potential. Cell.

[bib47] Siegel R.L., Miller K.D., Jemal A. (2016). Cancer statistics, 2016. CA. Cancer J. Clin..

[bib48] Souza-Fonseca-Guimaraes F., Huntington N.D. (2018). A new checkpoint for Natural Killer cell activation. Immunol. Cell Biol..

[bib49] Stromnes I.M., Hulbert A., Pierce R.H., Greenberg P.D., Hingorani S.R. (2017). T-Cell localization, activation, and clonal expansion in human pancreatic ductal adenocarcinoma. Cancer Immunol. Res..

[bib50] Tang Z., Kang B., Li C., Chen T., Zhang Z. (2019). GEPIA2: an enhanced web server for large-scale expression profiling and interactive analysis. Nucleic Acids Res..

[bib51] Teng M.W.L., Darcy P.K., Smyth M.J. (2011). Stable IL-10: a new therapeutic that promotes tumor immunity. Cancer Cell.

[bib52] Vinay D.S., Ryan E.P., Pawelec G., Talib W.H., Stagg J., Elkord E., Lichtor T., Decker W.K., Whelan R.L., Kumara H.M.C.S. (2015). Immune evasion in cancer: mechanistic basis and therapeutic strategies. Semin. Cancer Biol..

[bib53] Von Hoff D.D., Ervin T., Arena F.P., Chiorean E.G., Infante J., Moore M., Seay T., Tjulandin S.A., Ma W.W., Saleh M.N. (2013). Increased survival in pancreatic cancer with nab-paclitaxel plus gemcitabine. N. Engl. J. Med..

