# Supplementary material for: Targeting myeloid suppressive cells revives cytotoxic anti-tumor responses in pancreatic cancer
Source: iScience. 2022 Oct 9;25(11):105317. doi: 10.1016/j.isci.2022.105317 (PMC9615326; doi:10.1016/j.isci.2022.105317)
Supplement: Document S1. Figures S1–S5 and Tables S1 and S2 [file mmc1.pdf]

iScience, Volume *25*

Supplemental information

Targeting myeloid suppressive cells revives cytotoxic anti-tumor responses in pancreatic cancer

Dhifaf Sarhan, Silke Eisinger, Fei He, Maria Bergsland, Catarina Pelicano, Caroline Driescher, Kajsa Westberg, Itziar Ibarlucea Benitez, Rawan Humoud, Giorgia Palano, Shuijie Li, Valentina Carannante, Jonas Muhr, Björn Önfelt, Susanne Schlisio, Jeffrey V. Ravetch, Rainer Heuchel, Matthias J. Löhr, and Mikael C.I. Karlsson

Sarhan et. al. Figure S1.

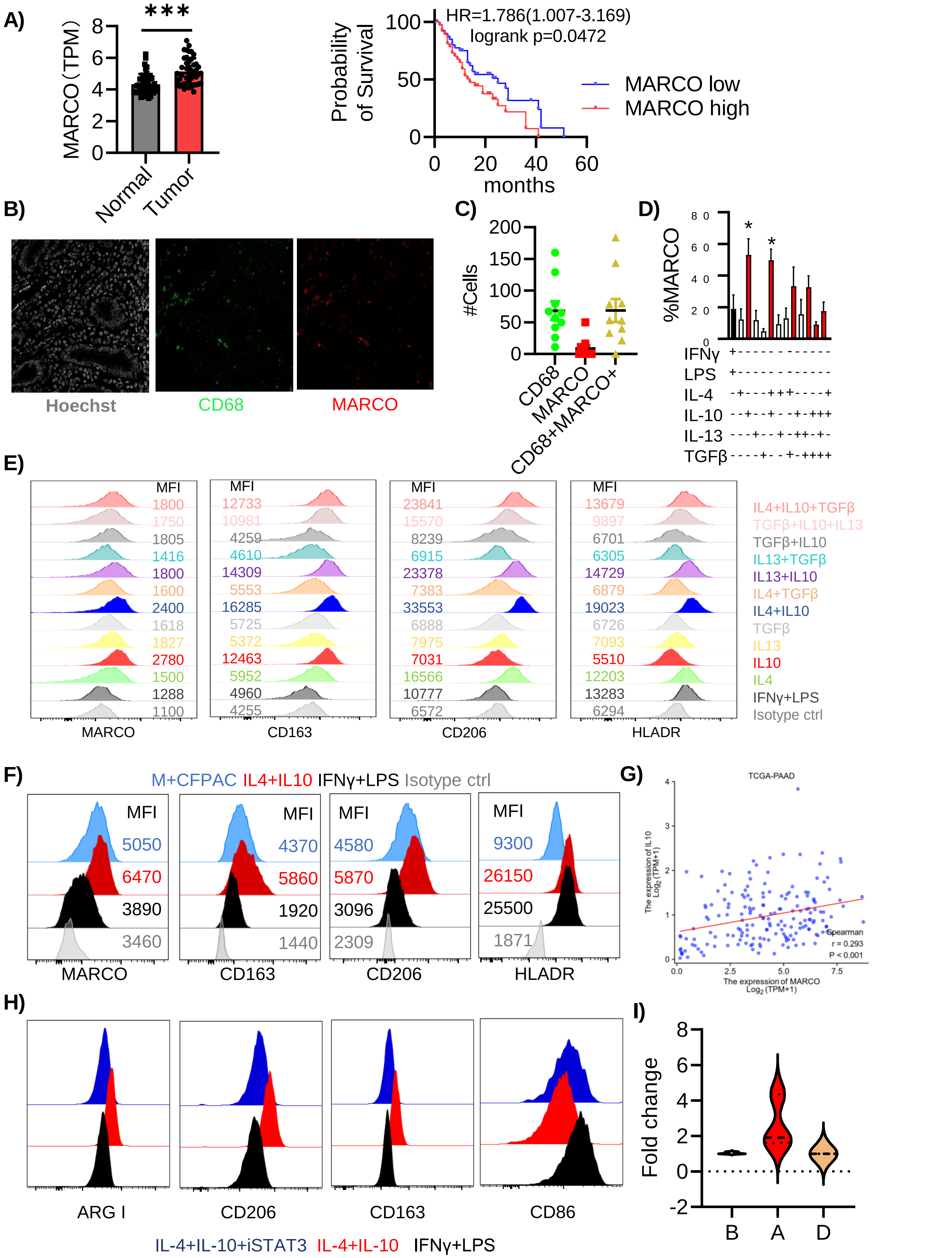

Figure S1. Characterization of human MARCO$^+$ myeloid cells in patients and *in vitro*, related to Figure 1 and Figure 2

A) Left panel: Analysis of mRNA expression of MARCO in 45 healthy individuals and 45 PDAC samples from the GEO data base (cohort ID GSE28735). Log_2, TPM+1 are shown. Right panel: Kaplan-Meier plot of PDAC patients (n=78) based on low versus high MARCO expression. Log-rank statistical analysis was used. **B)** and **C)** A representative single color of immunofluorescence staining and pooled quantitative data of different PDAC tumors are shown. Immunofluorescence staining of paraffin embedded pancreatic cancer PDAC sections (n=10). MARCO (red), CD68 (green), CD3, and Hoechst (gray) are shown. Quantification of number of (#) of CD68, MARCO+ and MARCO- macrophage areas in imaged PDAC sections was performed in ImageJ. Every dot represents an average of all imaged areas of one patient. **D)** Purified monocytes from HD (n=4-6) were differentiated in M-CSF and later polarized with combinations of different cytokines including; LPS+IFNγ (black bars), IL-4, IL-10 (red bars), IL-13, and TGFβ. Cells were assessed for %MARCO expression analyzed by flow cytometry. Statistical analyses were done using one-way ANOVA comparing all groups to IFNγ+LPS. *$p \leq 0.05$. Purified monocytes from HD were differentiated in M-CSF and later polarized with **E)** cytokine cocktails and **F)** conditioned with PC cell lines in transwell co-cultures and incubated overnight then analyzed for the expression of different pro vs. anti-inflammatory markers by flow cytometry. Representative histograms are shown. **G)** Correlation analysis of IL10 and MARCO gene expression in the TCGA PDAC cohort. **H)** Protein expression analyses by flow cytometry of ARG I, CD206, CD163, and CD86 in macrophages polarized as indicated in the presence or absence of the STAT3 small molecule inhibitor (iSTAT3). Representative histograms are shown of at least 4 independent experiments. **I)** Chromatin Immunoprecipitation assay (n=3) was performed to assess STAT3 binding to MARCO promotor (two expected binding regions A and D, and one negative region B). Detection of ChIP signal was done by qPCR and fold enrichment of amplified regions were calculated as Ct_{IgG}-Ct_{STAT3}.

Sarhan et. al. Figure S2.

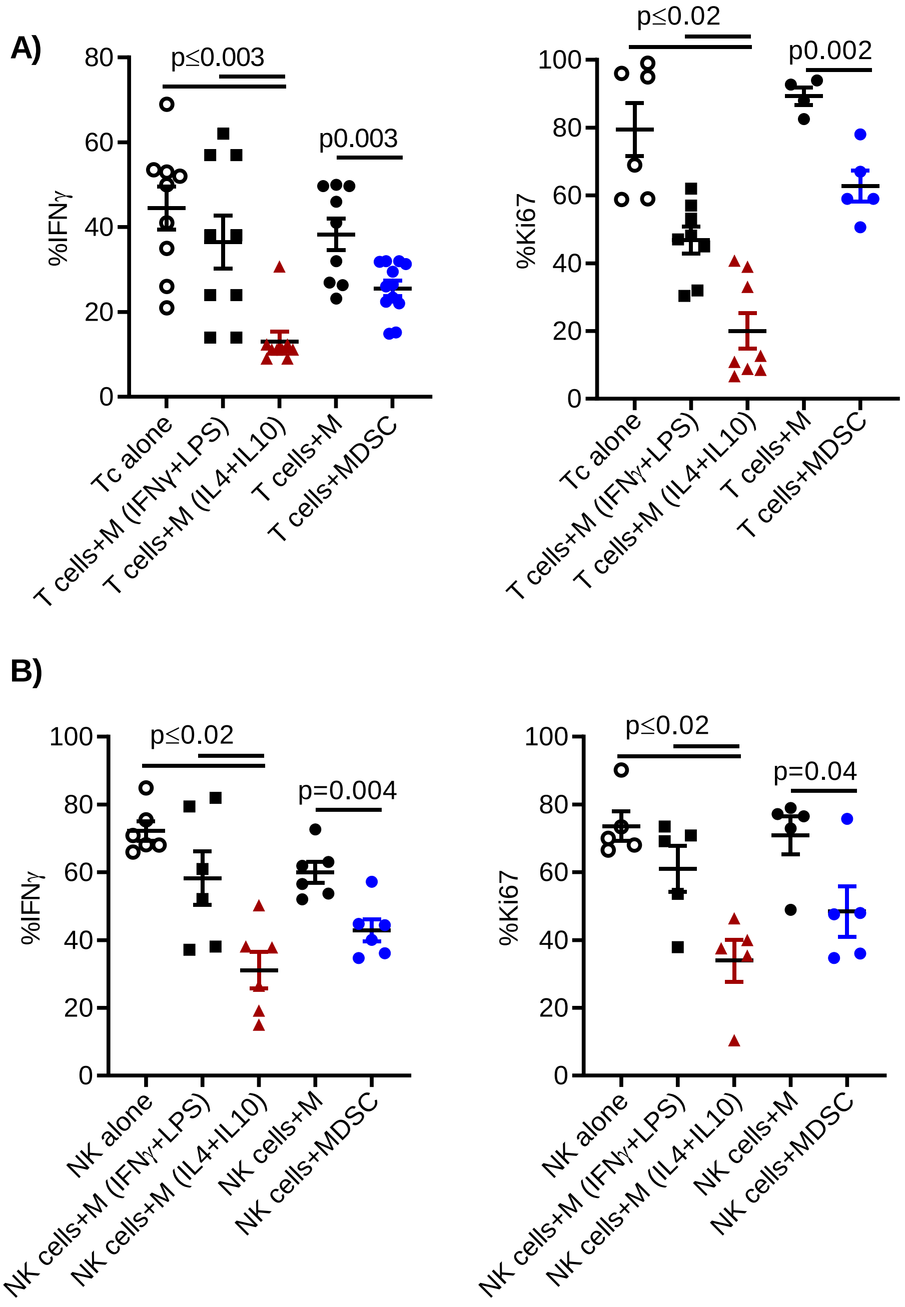

Figure S2. *In vitro* generated anti-inflammatory macrophages and MDSC are confirmed to suppress NK and T cells, related to Figure 4

Purified **A)** T or **B)** NK cells were co-cultured with macrophages, control monocytes, or MDSC for 3 days at a 1:1 ratio and evaluated for IFNγ production and proliferation (Ki67). T cell proliferation and IFNγ was assessed following stimulation using CD3/CD28 activation in a mixed lymphocyte reaction (MLR) and NK cells with IL-15. NK and T cell function was then evaluated following stimulation with PMA and ionomycin for 6 hours prior to staining. Pooled data are presented as mean±SEM and statistical analyses were performed using student paired T test.

Sarhan et. al. Figure S3.

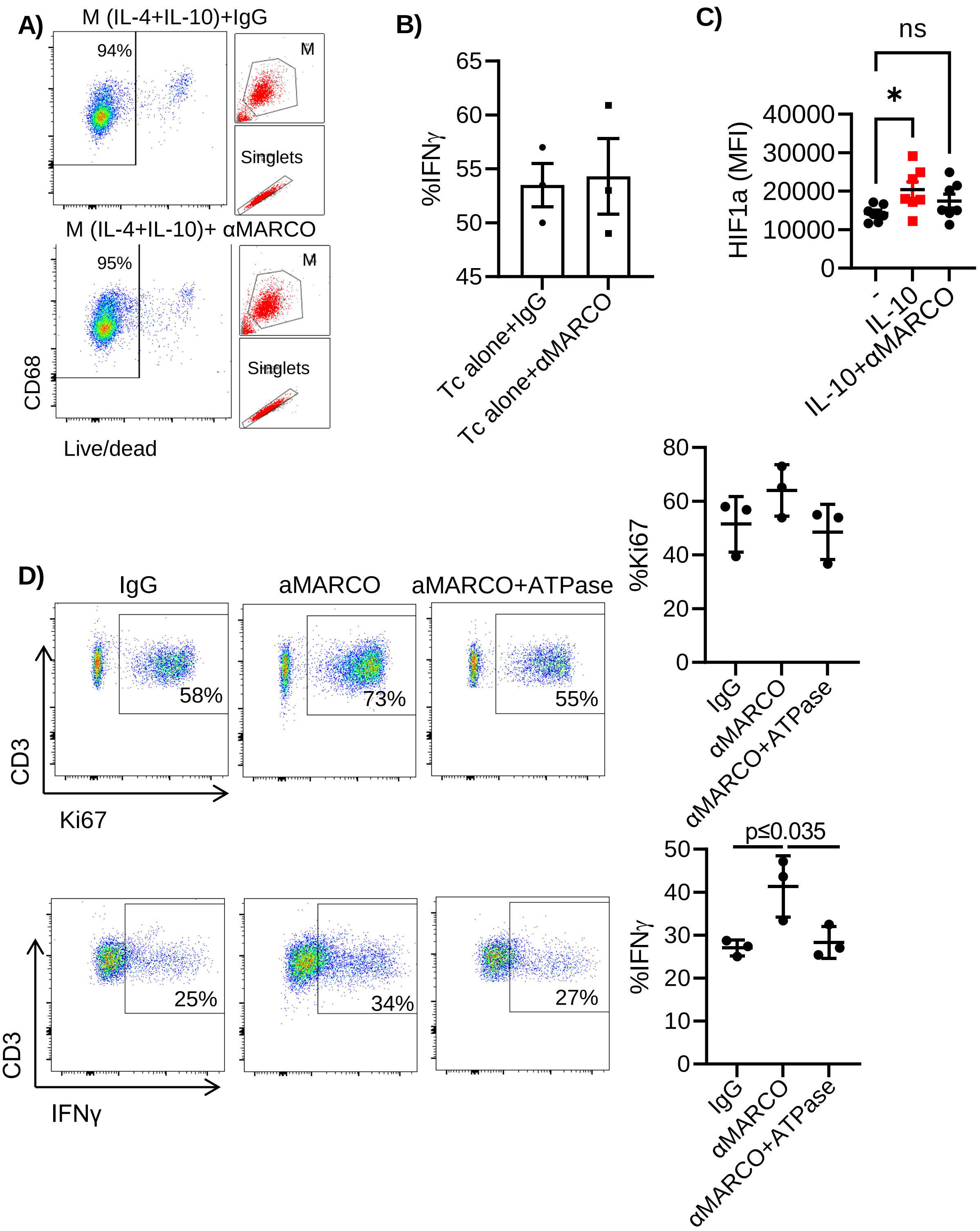

Figure S3. Anti-hMARCO treatment remodel myeloid cell signaling, related to Figure 5A, Figure 6, and Figure 7

A) Representative flow cytometery plots showing macrophage viability following polarization with IL-4+IL10 in the presence or absence of anti-MARCO antibodies. **B)** T cells cultured in the presence of IgG or anti-MARCO antibodies for 3 days and assessed for IFNγ production by Flow cytometry. Pooled data (n=3) are presented as mean±SEM. **C**) Monocytes were isolated from HD PBMC and cultured for 5 days in M-CSF and overnight polarized towards anti-inflammatory macrophages with IL-10 in the presence or absence of anti-MARCO antibodies, or left in M-CSF, and later assessed for HIF1α expression analyzed by flow cytometry. **D)** T cells (n=3) were co-cultured with anti-inflammatory macrophages at a 1:1 ratio ± anti-MARCO in the presence or absence of ATPase, for 3 days and assessed for proliferation (Ki67) and IFNγ production by flow cytometry. Representative flow cytometry plots and pooled data mean±SEM are shown and statistical analyses were performed using One-Way ANOVA.

Sarhan et. al. Figure S4.

A)

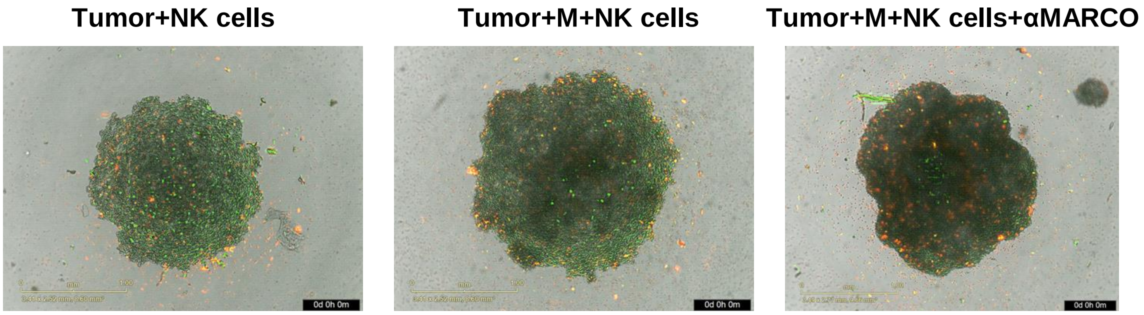

B)

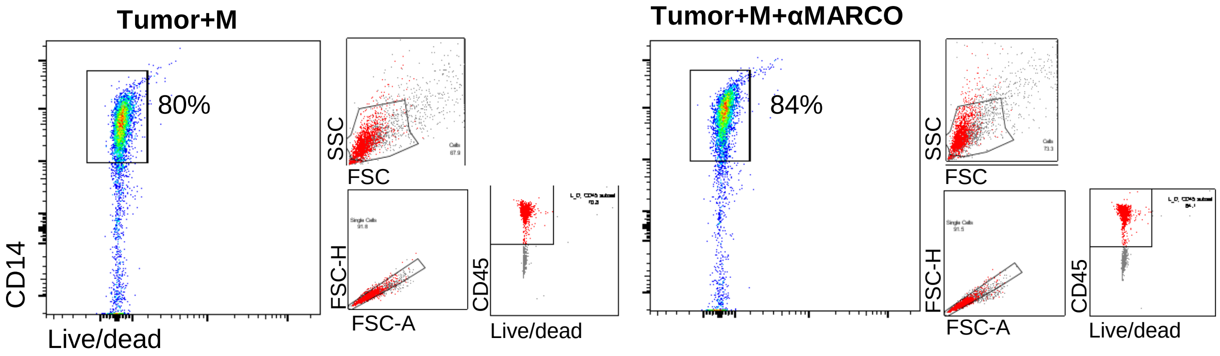

Figure S4. Anti-hMARCO treatment enable NK cell tumor infiltration and killing, related to Figure 7E

A) Representative images (n=7) showing NK cell infiltration (red) into PC tumor spheres with or without macrophages and in the presence of anti-MARCO treatment. **B)** Representative flow cytometery plots showing macrophage viability following 3D culture with tumors in the presence or absence of anti-MARCO antibodies.

Sarhan et. al. Figure S5.

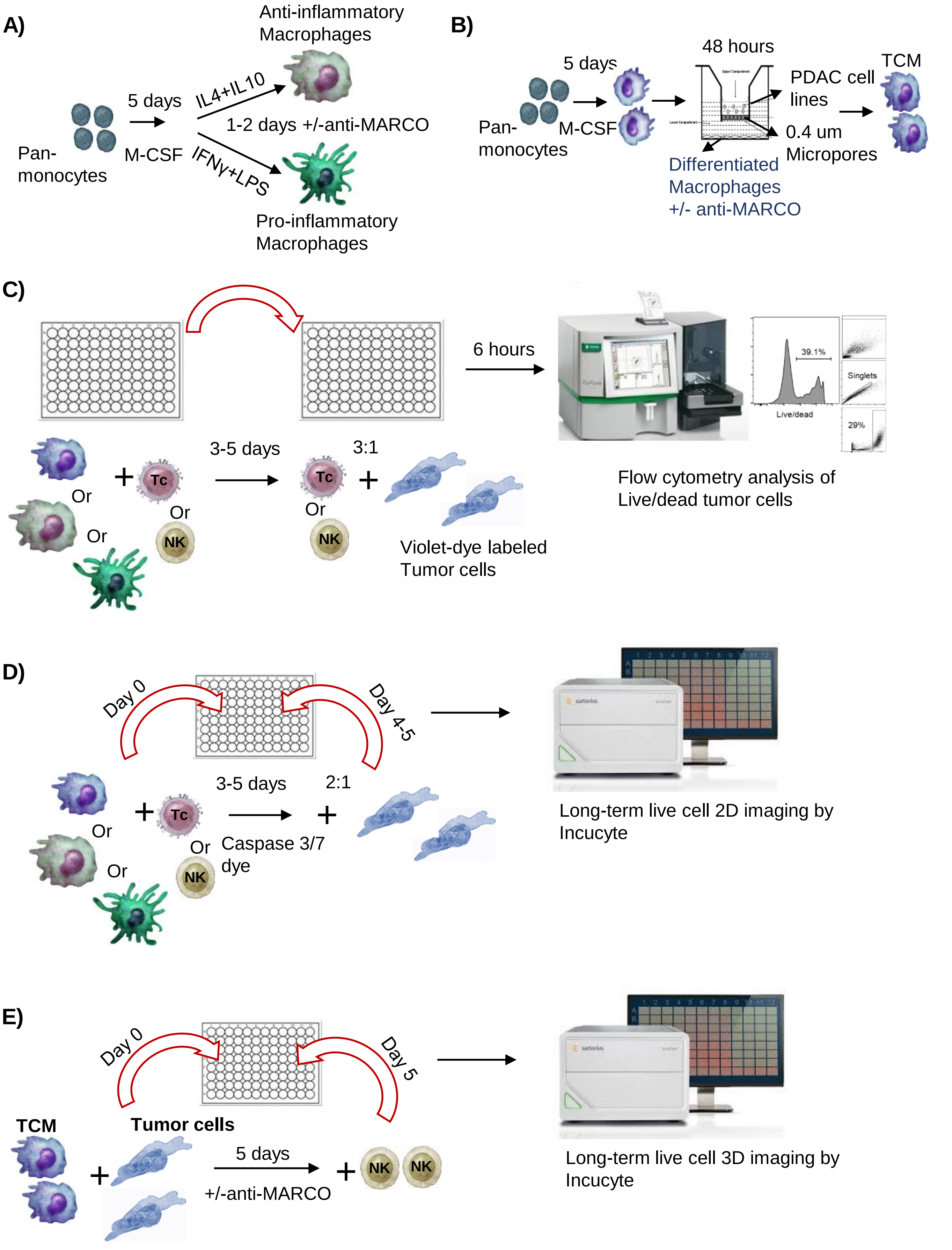

Figure S5. Methods, related to STAR Methods

Methodological scheme for generation of **A)** cytokine derived macrophages and **B)** tumor-conditioned macrophages (TCM). **C)** A schematic illustration of flow cytometry based killing assay. Schematic illustrations for long-term live cell **D)** 2D or **E)** 3D imaging by Incucyte system.

Table S1: Pancreatic cancer cell line characteristics

Cell line	Age	Sex	Derivation	Differentiation	Genotype
AsPC-1	62	Female	Ascites	Poor	$KRAS^{mut}$, $TP53^{mut}$
CFPAC-1	26	Male	Liver metastasis	Well	$KRAS^{mut}$,$TP53^{mut}$
Capan-2	56	Male	Primary tumor	Well	$KRAS^{mut}$, $TP53^{WT}$
PANC-1	56	Male	Primary tumor	Poor	$KRAS^{mut}TP53^{mut}$
BxPC-3	61	Female	Primary tumor	Moderate to poor	$KRAS^{WT}$, $TP53^{mut}$
MIA PaCa-2	65	Male	Primary tumor	Poor	$KRAS^{mut}$, $TP53^{mut}$
Capan-1	40	Male	Liver metastasis	Well	$KRAS^{mut}$, $TP53^{mut}$

Table S2. Primers used in the study

Primer	Forward	Reverse
**IL-12 p40**	CCAAGAACTTGCAGCTGAAG	TGGGTCTATTCCGTTGTGTC
**IL-1β**	GCAAGGGCTTCAGGCAGGCCGC G	GGTCATTCTCCTGGAAGGTCTGTGG GC
**TNF-α**	CTCTTCTCCTTCCTGATCGTGGCA	GTTGGATGTTCGTCCTCCTCACA
**MRC1**	TACAAAAAGGACAAACACCAAAA CC	TTGTAAATAACCCACCCATCTTCAG
**IL10**	ACATCAAGGCGCATGTGAACT	TGGCTTTGTAGATGCCTTTCTCTT
**IL1RN**	CTCAGCCAACACTCCTAT	TCCTGGTCTGCAGGTAA
**COX2**	TTCAAATGAGATTGTGGGA AAT	AGATCATCTCTGCCTGAGTA TCTT
**PDGFD**	CCCAGGAATTACTCGGTCAA	ACAGCCACAATTTCCTCCAC
**FN1**	CAACTCACTGACCTAAGCTT TGTTG	CGGTACCCAATAATGGTGGAA
**MARC O**	CAGCGGGTAGACAACTTCACT	TTGCTCCATCTCGTCCCATAG
**FIZZ1**	AGTGGTCCAGTCCACCACAC	AGTGTCAAAAGCCAAGGCAG
**GAPDH**	ACCATCATCCCTGCCTCTAC	CCTGTTGCTGTAGCCAAAT
**TIMP1**	CTTCTGGCATCCTGTTGTTG	GGTATAAGGTGGTCTGGTTG

**Primer name**	**Forward Primer**	**Reverse Primer**	**Within genomic region hg19**
MARCO_A	5´- AGGACATGTCTGATGCAT	5´- CATCACTATTTCTAGATC	chr2:119671593-119671936
MARCO_B	5´- ATGAGTGTTGAGCAATGTA	5´-TCACTTCACCTCTGGCTA	chr2:119697589-119697904
MARCO_D	5´- ACACAGTTGGCTCATCCCCA	5´- TGTGACCTTGAAGACCTGA	chr2:119731671-119731991
